# Transcriptome and Metabolome Analyses Reveal Potential Salt Tolerance Mechanisms Contributing to Maintenance of Water Balance by the Halophytic Grass *Puccinellia nuttalliana*

**DOI:** 10.3389/fpls.2021.760863

**Published:** 2021-10-29

**Authors:** Maryamsadat Vaziriyeganeh, Shanjida Khan, Janusz J. Zwiazek

**Affiliations:** Department of Renewable Resources, University of Alberta, Edmonton, AB, Canada

**Keywords:** *Puccinellia nuttalliana*, salt stress, RNA sequencing, metabolomics, *de novo* assembly

## Abstract

Elevated soil salinity exacerbated by human activities and global climate change poses serious threats to plant survival. Although halophytes provide many important clues concerning salt tolerance in plants, some unanswered questions remain to be addressed, including the processes of water and solute transport regulation. We performed high-throughput RNA-sequencing in roots and metabolome characterizations in roots and leaves of *Puccinellia nuttalliana* halophytic grass subjected to 0 (control) and 150 mM NaCl. In RNAseq, a total of 31 Gb clean bases generated were *de novo* assembled into 941,894 transcripts. The *PIP2;2* and *HKT1;5* transcript levels increased in response to the NaCl treatment implying their roles in water and ion homeostasis. Several transcription factors, including *WRKY39*, *DEK3*, *HY5*, and *ABF2*, were also overexpressed in response to NaCl. The metabolomic analysis revealed that proline and dopamine significantly increased due to the upregulation of the pathway genes under salt stress, likely contributing to salt tolerance mechanisms. Several phosphatidylcholines significantly increased in roots suggesting that the alterations of membrane lipid composition may be an important strategy in *P. nuttalliana* for maintaining cellular homeostasis and membrane integrity under salt stress. In leaves, the TCA cycle was enriched suggesting enhanced energy metabolism to cope with salt stress. Other features contributing to the ability of *P. nuttalliana* to survive under high salinity conditions include salt secretion by the salt glands and enhanced cell wall lignification of the root cells. While most of the reported transcriptomic, metabolomics, and structural alterations may have consequences to water balance maintenance by plants under salinity stress, the key processes that need to be further addressed include the role of the changes in the aquaporin gene expression profiles in the earlier reported enhancement of the aquaporin-mediated root water transport.

## Introduction

Soil salinity is a challenging problem that affects plants in many natural ecosystems and areas affected by human activities. The concerns of primary salinization due to the factors such as seawater intrusion and volcanic bedrock weathering (Alfarrah and Walraevens, 2018; Mirzavand et al., 2020) have been grossly aggravated by secondary salinization due to anthropogenic manipulation of the hydrologic cycle (Herbert et al., 2015). Rising mean temperatures in many parts of the world enhance secondary salinization by increasing evapotranspiration, leading to salt buildup in the soil (Pankova and Konyushkova, 2014; Okur and Örçen, 2020). According to the 2013 estimate, current annual economic losses due to salt-induced land degradation could be as much as $27 billion (Shahid et al., 2018).

High salinity areas are dominated by salt-tolerant halophytic plant species, which can survive salt concentrations exceeding 300 mM NaCl (Flowers and Colmer, 2008; Al Hassan et al., 2016). Halophytes constitute only a tiny fraction of all higher plant species, comprising approximately 1% of the world’s flora (Sairam and Tyagi, 2004). Halophytic plants have developed unique structural and physiological strategies to cope with salt (Shabala et al., 2014; Flowers et al., 2015). Despite the ability of halophytic plants to tolerate high salt concentrations, most studies on salt effects and salt tolerance in plants have been carried out in glycophytes since they include most of the economically important plant species. However, with the growing salinity concerns, halophytes are gaining more attention due to their successful salt tolerance strategies.

Numerous efforts have been made to understand salinity effects on plants and the mechanisms of plant salt resistance for crop improvement and development of vegetation management strategies in salt-affected areas (Bushman et al., 2016; Nikalje et al., 2017; Aliakbari et al., 2021). Salinity affects all stages of plant development including seed germination, vegetative and reproductive growth (Shrivastava and Kumar, 2015). It affects plants through a combination of osmotic factors, direct ion toxicity, nutrient imbalance, and reactive oxygen species (ROS) accumulation (Munns and Tester, 2008; Flowers et al., 2015). Plant survival in soils with elevated salinity requires an efficient water-transporting system, which can overcome osmotic effects of salt and direct ion toxicity that affect water transport in salt-sensitive plants.

Water transport is partly regulated by the aquaporins (Voicu et al., 2009; Groszmann et al., 2017). Aquaporin proteins form water channels, which facilitate the passive transport of water and a range of neutral solutes and gasses across the cell membranes (Maurel et al., 2008; Zwiazek et al., 2017; Tan et al., 2018). It has been demonstrated that the aquaporin-mediated water transport is rapidly inhibited in glycophytic plants by NaCl concentrations as low as 10 mM (Lee and Zwiazek, 2015). However, cell hydraulic conductivity was enhanced in *P. nuttalliana* by 50 and 150 mM NaCl (Vaziriyeganeh et al., 2018). Subsequent study showed that Na^+^ was the factor responsible for the enhancement of aquaporin-mediated root water transport in this halophytic grass resulting in higher cell hydraulic conductivity. However, the exact processes contributing to this enhancement, including those at the molecular level, remain unknown.

Plant molecular responses to NaCl involve complex gene regulatory networks that may directly and indirectly affect the function of aquaporins and, ultimately, plant water transport. As soon as plants perceive salt stress, signaling molecules such as ROS, Ca^2+^, and various phytohormones are activated. Transcription factor (TF) families have important roles in plant responses to abiotic stress. TFs such as MYB, NAC, WRKY, bHLH, bZIP, AP2/ERF, MYB, and ERF/DREB may be involved in plant stress responses (Baillo et al., 2019; Das et al., 2019). The salt overly sensitive (SOS) signal transduction cascade consisting of SOS3 (CBL calcium sensor), SOS2 (CIPK protein kinase), and SOS1 (Na^+^/H^+^ antiporter) genes encode proteins that are involved in Na^+^ extrusion (Zhu et al., 1998; Ji et al., 2013) to regulate plant ion homeostasis. Salt stress triggers cytosolic Ca^2+^ signaling (Hasegawa et al., 2000; Parre et al., 2007). Ca^2+^ binds to SOS3 and forms a SOS3-SOS2 complex by activating SOS2 leading to the activation of the downstream SOS1, a Na^+/^H^+^ antiporter. Several other ion transporters function as pumps for the sequestration of Na^+^ into vacuoles of both roots and shoots (Nikalje et al., 2017). Exposure of plants to salt also triggers changes in the aquaporin expression (Lee and Zwiazek, 2015; Braz et al., 2019). However, gene expression data alone cannot unravel complex responses that are triggered by salt and lead to altered hydraulic responses in plants.

Since salinity tolerance is a complex trait that involves multi-gene responses only a comprehensive study approach involving different methods can provide the best platform for the efforts aimed at improvement of salt tolerance in plants. Recent advances in metabolomics and transcriptomics (Cavill et al., 2016; Ren et al., 2019) have created an opportunity to examine a wide spectrum of these complex plant responses contributing to salt tolerance. While transcriptomics can provide useful information concerning the functional elements of the genome and associated pathways related to salt stress, combining the transcriptomics and metabolomics data can be a powerful tool to better characterize the molecular and functional traits involved in the salt tolerance processes.

In the present study, we exposed the halophytic northern grass *Puccinellia nuttalliana* to the 150 mM NaCl treatment for 6 days and performed root transcriptome analysis using RNA-Seq and root and leaf metabolome analysis using tandem mass spectrometry (DI-MS/MS) coupled with a liquid chromatography-tandem mass spectrometry (LC-MS/MS) to understand the mechanisms underlying salt tolerance in these halophytic plants. We were especially interested in unraveling the processes enabling *P. nuttalliana* to maintain the functionality of aquaporin-mediated water transport under salt stress conditions that were earlier reported (Vaziriyeganeh et al., 2018). We hypothesized that ion and osmotic homeostasis combined with changes in the aquaporin gene profile are the key elements involved in water balance maintenance under salinity conditions. We also followed these analyses with the measurements of plant growth parameters, root and leaf structure, and tissue ion concentrations to obtain a more complete view of the processes contributing to salt tolerance in this halophytic plant.

## Materials and Methods

### Growth Conditions and NaCl Treatment

Seeds of *P. nuttalliana* (Schult.) Hitchc. were surface-sterilized for 2 min with 70% ethanol followed by 5% sodium hypochlorite for 5 min. The sterilized seeds were rinsed in autoclaved water and spread on plates containing half-strength Murashige and Skoog (MS) solid medium (Murashige and Skoog, 1962) with no added sugar or hormones. After 3 days of germination, the seedlings were transferred to plastic containers filled with horticultural soil and grown in the controlled-environment growth room with 22/18°C (day/night) temperature, 16-h photoperiod with 300 μmol m^–2^ s^–1^ photosynthetic photon flux density, and 50–60% relative humidity. Plants were fertilized once a week with half-strength modified Hoagland’s solution (Epstein, 1972) and were watered twice a week to runoff. After 8 weeks of growth in the soil, 16 seedlings were removed from pots, their roots washed, and placed in four 11 L containers filled with aerated 50% Hoagland’s mineral solution. The containers with seedlings were placed in a controlled-environment growth room with the growth conditions as described above and grown for 1 week prior to the commencement of NaCl treatment. The plants were treated for 6 days with 0 (control) and 150 mM NaCl, which was added gradually to the nutrient solution, in 50 mM increments over the course of 1 day, to reduce osmotic shock. The NaCl treatment concentration and duration was selected based on the previous study, (Vaziriyeganeh et al., 2018) that showed an enhancement of growth and root cell hydraulic conductivity in *P. nuttalliana*, while a significant decrease in these parameters was observed in *P. pratensis*. The experiment was arranged in the randomized complete block design with plants placed in three replicated containers per treatment (control and 150 mM NaCl). Four (*n* = 4) and three (*n* = 3) plants were randomly taken from the three replicated containers for metabolomic analysis and transcriptome sequencing, respectively, and six plants (*n* = 6) for dry weight determinations and elemental analysis.

### Plant Dry Weights and Tissue Elemental Analysis

After 6 days of treatments, shoot and root dry weights were determined in six seedlings per treatment (*n* = 6). Plants were harvested, and their shoots and roots separated and placed in an oven at 70°C for 72 h and weighed. The total plant dry weights were obtained by combining shoot and root dry weights. For the analyses, root and shoot samples (0.2 *g* dry weight) of six plants per species (*n* = 6) were collected after 6 days of treatments. To determine shoot and root concentrations of Na, K, and Cl, the samples from six plants per treatment (*n* = 6) were digested with 70% HNO_3_ and heated for 10 min at 185°C in a microwave oven (Mars 5 Microwave Accelerated Reaction System, CEM, Matthews, NC, United States). The extracts were diluted with Milli-Q water, filtered, and analyzed with the inductively coupled plasma – optical emission spectrometer (iCap 6000, Thermo Fisher Scientific Inc, Waltham, MA, United States). Tissue chloride was analyzed in hot water extracts using the EPA 325.2 ferrothiocyanate method (US Environmental Protection Agency 1983) with the Thermo Gallery plus Beermaster Auto analyzer (Thermo Fisher Scientific, Vantaa, Finland). The elemental analyses were carried out in the Natural Resources Analytical Laboratory of the University of Alberta, Edmonton, Canada.

### RNA Extraction

Total RNA was extracted from the roots of *P. nuttalliana* using the RNeasy Plant Mini Kit (QIAGEN, Venlo, Netherlands). The quality of total RNA was determined by gel electrophoresis, and the concentration of the extracted total RNA was determined using a NanoDrop 2000c spectrophotometer (Thermo Fisher Scientific Inc., Waltham, MA, United States). The RNA quality control [RNA concentration, RNA Integrity Number (RIN) value, 28S/18S, and the fragment length distribution] was performed using a 2100 Bioanalyzer (Agilent, Mississauga, ON, Canada). We ensured that the RIN values were greater than 7.0.

### cDNA Library Preparation and RNA Sequencing

The mRNA was fragmented, and then poly(A) + mRNA was isolated using oligo dT. Double-stand cDNA was synthesized from the fragmented RNA by N6 random primer. The synthesized cDNA was subjected to end-repair and then was 3′ adenylated. Successively, DNA oligonucleotides adaptors were ligated to the ends of these 3′ adenylated cDNA fragments. The ligation products were purified, and several rounds of PCR amplification were performed to enrich the purified cDNA template. The double-strand PCR products were denatured by heat, and the single-strand DNA was cyclized by splint oligo and DNA ligase. The sequencing of each cDNA library was carried out in the BGISEQ-500 system with the paired-end sequencing length of 100 bp according to the manufacturer’s instructions at the Beijing Genomics Institute (BGI-Shenzhen, China).

### Sequencing Reads Filtering, *de novo* Assembly and Functional Annotation

The sequencing reads containing low quality, adaptor-polluted, and high content of unknown base (N) reads were processed to be removed before downstream analyses. The raw data filtering component statistics and clean reads quality metrics are provided in the Supplementary Table 1. We used Trinity (Grabherr et al., 2011) to perform *de novo* assembly with clean reads. We used Tgicl (Pertea et al., 2003) on cluster transcripts to remove abundance and obtain unigenes. Functional annotation of the unigenes was performed by a BLASTx search with an *E*-value of 10-5 against seven protein databases included non-redundant (NR) protein database, NR nucleotide sequence (Nt) database, Gene ontology (GO), euKaryotic Orthologous Group database (KOG), and Kyoto Encyclopedia of Genes and Genomes protein database (KEGG), SwissProt and InterPro. Additionally, Blast2GO (Conesa et al., 2005) was used to obtain the GO. We calculated the ratio of different unigene annotations based on the NR database functional annotation results.

### Unigene Simple Sequence Repeats Detection

We used MISA version: v1.0 using the parameters: 1-12,2-6,3-5,4-5,5-4,6-4 100 150 to detect simple sequence repeats (SSRs) in unigenes, then designed primers for each SSR with the Primer3 software^1^ using the default parameters.

### Unigene Expression and Differentially Expressed Genes Detection

We used Bowtie2 (Langmead and Salzberg, 2012) version: v2.2.5 to map clean reads to unigenes and then calculate the gene expression level with RSEM (Li et al., 2011) version: v1.2.12 using the default parameters. The Fragments per kilobase per transcript per million mapped reads (FPKM) method was used (Trapnell et al., 2010) to compare the gene expression differences among different samples. We detected Differentially Expressed Genes (DEGs) with DEseq2 using the parameters: fold change ≥ 2.00 and adjusted *P* value ≤ 0.05. GO and KEGG pathway and enrichment analysis were performed on the DEGs.

### Validation of Expression Changes by Quantitative Real-Time PCR

To confirm the gene expression data obtained from the RNA-seq data, a subset of DEGs was validated by quantitative real-time PCR (qRT-PCR). Four plants per treatment and three technical replications were used. The genes included *HKT1;5* (Unigene42482_All), *PIP2;2* (Unigene159054_All), *TIP4;4* (Unigene43183_All), *WRKY17* (CL11112.Contig4_All), *DUF4220* (Unigene37730_All), *CBL10* (Unigene160802_All), *MYB77* (Unigene25779_All), and *HAK9* (CL646.Contig7_All). Roots were washed using distilled water and blot-dried with a paper towel. The collected tissues were immediately flash-frozen in liquid nitrogen and stored in −80°C freezer until used. The roots were homogenized by grinding in liquid nitrogen with a pestle and mortar and total RNA was extracted from roots using QIAGEN RNeasy Plant Mini Kit. The RNA concentration and purity were assessed using a Thermo Scientific^TM^NanoDrop^TM^ One Microvolume UV-Vis Spectrophotometer (Thermo Scientific^TM^). RNA quality was also checked on a 1% (w/v) agarose gel. The removal of genomic DNA contamination and first-strand cDNAs were generated using the QuantiTect Reverse Transcription Kit (Qiagen, CA, United States) with 500 μg total RNA according to the manufacturer’s instructions. The qRT-PCR was performed using SYBR Green I dye reagent in an Applied Biosystems 7500 Fast system with 10-fold diluted cDNA. The relative expression of all genes was calculated using the 2^–Δ^ ^Δ^ ^*CT*^ method (Livak and Schmittgen, 2001). We performed melting curve analysis and gel electrophoresis for the specificity of the PCR amplification product for each primer pair. Two reference genes, *ACT* (Actin) and *ADP* (ADP-ribosylation factor 1) were used for normalization. The primers used for the qRT PCR analysis are listed in Supplementary Table 2. For validation of primers, we used serial dilutions of the cDNA to create a standard curve by plotting the log value of starting templet quantity against the Ct values obtained for each dilution. Amplification efficiency (E) was calculated using slope from linear regression of the standard curve according to the equation, *E* = (10^–1/slope^–1) × 100 and are expressed as percentage (Supplementary Table 2).

### DI/LC-MS/MS

The individual freeze dried and ground plant root and leaf tissue samples were placed in screw vials and sent to The Metabolomics Innovation Centre, AB, Canada for further sample preparation and analysis. A targeted quantitative metabolomics approach was applied to analyze root and shoot samples using a combination of direct injection (DI) mass spectrometry and a reverse-phase LC-MS/MS custom assay. This custom assay, in combination with an ABSciex 4000 QTrap (Applied Biosystems/MDS Sciex) mass spectrometer, was used for the targeted identification and quantification of different endogenous metabolites including amino acids, acylcarnitines, biogenic amines and derivatives, organic acids, glycerophospholipids, and sugars (Foroutan et al., 2009, 2020). The method combines the derivatization and extraction of analytes, and the selective mass-spectrometric detection using multiple reaction monitoring pairs. Isotope-labeled internal standards and other internal standards were used for metabolite quantification. The custom assay contained a 96 deep-well plate with a filter plate attached with sealing tape, and reagents and solvents used to prepare the plate assay. First 14 wells were used for one blank, three zero samples, seven standards and three quality control samples. For all metabolites except organic acid, samples were thawed on ice and were vortexed and centrifuged at 13,000 × *g*. Each 10 μL pathway enrichment analysis was performed by entering the differential metabolites in the Pathway Analysis module of MetaboAnalyst 5.0 (https://www.metaboanalyst.ca). Sample was loaded onto the center of the filter on the upper 96-well plate and dried in a stream of nitrogen. Subsequently, phenyl-isothiocyanate was added for derivatization. After incubation, the filter spots were dried again using an evaporator. Extraction of the metabolites was then achieved by adding 300 μL of extraction solvent. The extracts were obtained by centrifugation into the lower 96-deep well plate, followed by a dilution step with the MS running solvent. For the organic acid analysis, 150 μL of ice-cold methanol and 10 μL of isotope-labeled internal standard mixture were added to 50 μL of each sample for overnight protein precipitation. Then it was centrifuged at 13,000 × *g* for 20 min. 50 μL of supernatant was loaded into the center of wells of a 96-deep well plate, followed by the addition of 3-nitrophenylhydrazine reagent. After incubation for 2 h, BHT stabilizer and water were added before LC-MS injection. Mass spectrometric analysis was performed using an ABSciex 4000 Qtrap^®^ tandem mass spectrometry instrument (Applied Biosystems/MDS Analytical Technologies, Foster City, CA, United States) equipped with an Agilent 1260 series UHPLC system (Agilent Technologies, Palo Alto, CA, United States). The samples were delivered to the mass spectrometer by the LC method followed by the DI method.

### Metabolome Data Analysis

Metabolites were searched using Analyst software (V.1.6.2) from AB SCIEX (Concord, ON, Canada). Principle component analysis (PCA) and statistical analyses were performed using MetaboAnalyst 5.0 (Pang et al., 2021). Pathway enrichment analysis was performed by entering the differential metabolites in the Pathway Analysis module of MetaboAnalyst 5.0 (https://www.metaboanalyst.ca). Venn diagrams were created using Venny 2.0.2 interactive tool (Oliveros, 2007–2015).

### Root Anatomy and Leaf Morphology

Distal root segments (*n* = 5) from each treatment were prepared for microscopy according to Roschzttardtz et al. (2009). Distal 3–5-cm root segments were fixed in formalin-acetic acid-alcohol (FAA) solution. After fixation, the root segments were dehydrated in an ethanol series and placed in toluene. Fixed root segments were embedded in paraffin and sectioned with a microtome (model RM2125 RTS, Leica; Solms, Germany). The sections were mounted on slides and examined under light microscope (Olympus SZ61 TR Stereo Light Microscope with SeBaCam 5.1MP Camera). Lignin auto fluorescence was visualized using green light (Filter Wheel Setting = 3, Green Image, Leica Filter cube: I3), following UV excitation at 330 nm to 380 nm with a fluorescent microscope (Carl Zeiss; Jena, German; Donaldson and Radotic, 2013). The light intensity of lignification was determined with Image J software^2^ (*n* = 5 images per group) and quantified as previously described (Yamaguchi et al., 2010).

### Sample Preparation for Scanning Electron Microscopy

Approximately 5-mm-long segments were excised from the control *P. pratensis* and *P. nuttalliana* plants and from plants treated for 6 days with 150 mM NaCl. The leaf samples were immediately placed in the FAA fixative and dehydrated using a series of increasing ethanol concentrations followed by hexamethyldisilazane and air-dried. The samples were then placed on metal stubs, attached with a double-sided carbon tape, sputter-coated with Au/Pd, and mounted on the SEM stubs. The samples were viewed using the ZEISS EVO 10 Scanning Electron Microscope (Carl Zeiss Microscopy, Köln, Germany) at the University of Alberta Microscopy Facility.

## Results

### Plant Dry Weights and Na^+^, K^+^, and Cl^–^ Tissue Concentrations

**FIGURE 1:**
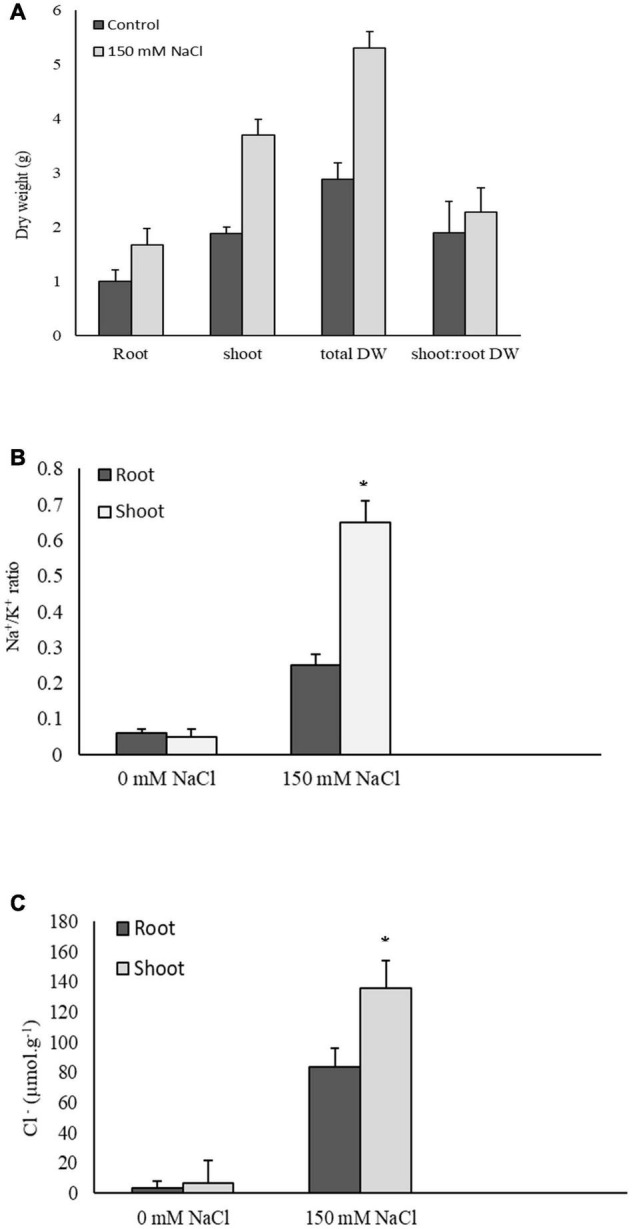
Root, shoot, and total dry weights and shoot: root dry weight ratios in *Puccinellia nuttalliana* treated with 0 mM and 150 mM NaCl for 6 days. Means (*n* = 6) ± SE are shown **(A)**, Na^+^/K^+^ ratios **(B)**, and Cl^–^ concentrations **(C)** in roots and shoots of *Puccinellia nuttalliana* treated with 0 mM and 150 mM NaCl for 6 days in hydroponic system. Asterisks above the bars indicate significant differences (*p* ≤ 0.05) between treatments as determined by the Tukey’s test. Means (*n* = 6) + SE are shown.

The 6-day treatment with 150 mM NaCl had no significant effect on the root, shoot, and total plant dry weights. Similarly, there was no change in the shoot:root dry weight ratios due to the NaCl treatment (Figure 1A). *P. nuttalliana* accumulated less K^+^ and more Na^+^ in roots compared with shoots resulting in about three-fold higher Na^+^/K^+^ ratio in roots than shoots under salt stress (Figure 1B). The concentration of Cl^–^ in shoots relative to roots was six times higher under NaCl treatment (Figure 1C).

### Sequencing and *de novo* Assembly

Transcriptome sequencing generated 52.47 million paired-end reads per sample and in total about 30.91 Gb clean bases. Raw reads were filtered for low-quality, adaptor-polluted, and high content of unknown base (N) reads. The statistics of the assembled sequence are provided in Table 1 and Trinity (1) based *de novo* assembly statistics from each assembly are presented in Table 1. The raw reads were deposited in the Sequence Read Archive of the National Center for Biotechnology Information database. The BioSample accessions are Pnu_RNAseq_Control (SRX8289102) and Pnu_RNAseq_Treated (SRX8289103).

**TABLE 1:** Output statistics for *Puccinellia nuttalliana* transcriptome sequencing and *de novo* assembly Trinity statistics for quality metrics of transcripts of each *P. nuttalliana* sample.

Filter summary						
**Sample**	**Total raw reads (M)**	**Total clean reads (M)**	**Total clean bases (Gb)**	**Clean reads Q20 (%)**	**Clean reads Q30 (%)**	**Clean reads ratio (%)**
C1	52.47	51.69	5.17	97.94	91.46	98.52
C2	52.47	51.49	5.15	97.96	91.61	98.12
C3	52.47	51.56	5.16	98.08	91.94	98.26
T1	52.47	51.38	5.14	97.98	91.84	97.92
T2	52.47	51.34	5.13	97.69	90.73	97.84
T3	52.47	51.56	5.16	97.86	91.41	98.25
***De novo* assembly Trinity statistics**						
**Sample**	**Total number**	**Mean length**	**N50**	**N70**	**N90**	**GC (%)**
C1	180284	565	873	445	232	46.9
C2	187042	576	886	465	237	46.78
C3	192381	587	914	477	240	46.1
T1	152132	575	929	459	230	49.15
T2	108930	691	1228	635	261	49.7
T3	121125	598	961	512	240	50.33

*C = control, *T* = 150 mM NaCl treatment, and 1–3 = replications.*

### Functional Annotation of Unigenes and Unigene Simple Sequence Repeat Detection

**FIGURE 2:**
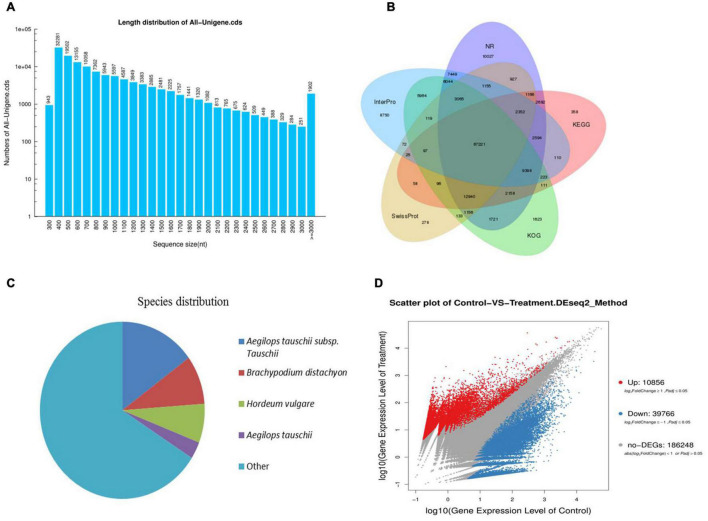
The length distribution of unigenes of *Puccinellia nuttalliana* transcriptome **(A)**, venn diagram showing functional annotation between NR, KOG, KEGG, Swissprot, and Interpro databases **(B)**, distribution and frequency of non-redundant (NR) annotated species **(C)**, scatter plot of differentially expressed genes (DEGs) in roots of NaCl-treated *Puccinellia nuttalliana* plants compared to the controls. Red color represents the up-regulated genes, blue color represents the down-regulated genes, gray color represents the non-significant differential genes **(D)**.

**FIGURE 3:**
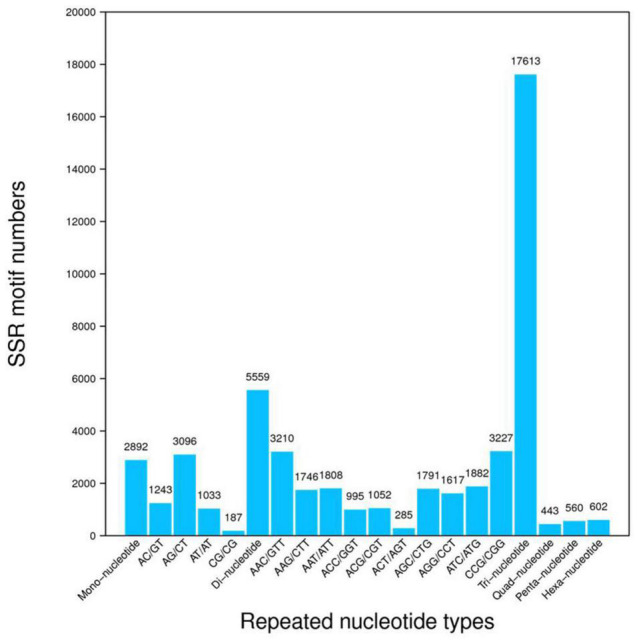
Frequency distribution of simple sequence repeats (SSR). The *x*-axis indicates the type of SSR and the *y*-axis indicates the number of SSR.

After assembly, a total of 242,833 unigenes were identified. The clustering quality metrics are shown in Supplementary Table 3, and the unigene length distribution is shown in Figure 2A. All unigenes were blast searched against the public databases, including non-redundant protein (Nr) database, non-redundant nucleotide (Nt) database, InterPro, Swiss-Prot, GO, KEGG, and Clusters of Orthologous Groups (KOG). The number and percentage of unigenes annotated by each database are summarized in Table 2. Results of functional annotation showed that 156,214 (64.33%) of 242,833 unigenes were successfully annotated by the databases. We used the Venn diagram to show the annotation results of NR, KOG, KEGG, SwissProt, and InterPro in Figure 2B. The Venn diagram showed that all five databases simultaneously annotated 67,221 unigenes. In functional annotation, 54.38% aligned unigenes in the NR database were used to calculate their distribution and frequency in different species. Based on the NR database, ∼34% of unigenes matched sequences from only three grass species including *Aegilops tauschii* (18.03%), *Brachypodium distachyon* (8.92), and *Hordeum vulgare* (7.11%; Figure 2C). The unigenes annotated by the Nr database were assigned into three gene sub-ontologies: biological process (26 subcategories), cellular component (16 subcategories), and molecular functions (14 subcategories; Supplementary Figure 1) using the Blast2GO program (Conesa et al., 2005). SSRs or microsatellites are short, tandemly repeated DNA motifs used to evaluate population genetic diversity and structure and genetic linkage mapping. *De novo* screening of large sets of SSR detection was performed for *P. nuttalliana*. The size summary of SSR is shown in Figure 3 and the details of the primers are provided in The Supplementary Data Sheet 1. Trinucleotide repeats were predominant (17,613). Five and six tandem repeats were found for 560 and 602 motifs, respectively. The prominent trinucleotide repeat was identified as CCG/CGG (3,227) and AAC/GTT (3,210).

**TABLE 2:** Annotation summary for unigenes of *Puccinellia nuttalliana* using public databases.

Values	Total	Nr	Nt	SwissProt	KEGG	KOG	InterPro	GO	Intersection	Overall
Number	242,833	132,055	65,723	90,860	101,589	112,069	114,638	77,665	23,491	156,208
Percentage	100%	54.38%	27.07%	37.42%	41.83%	46.15%	47.21%	31.98%	9.67%	64.33%

### Validation of RNA-Seq Results by Quantitative Real-Time PCR

**FIGURE 4:**
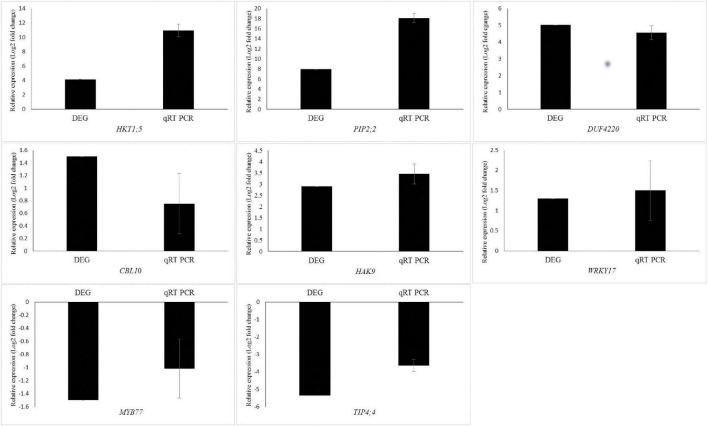
Validation of transcriptional changes of eight randomly selected genes from the DEGs using qRT-PCR of *Puccinellia nuttalliana* under NaCl stress. Values are means + SE of four biological replicates. Each biological replicate is comprised of three technical replicates.

To validate the RNA-Seq transcriptome profiling, eight DEGs including *HKT1;5*, *PIP2;2*, *TIP4;4*, *WRKY17*, *DUF4220*, *CBL10*, *MYB77*, and *HAK9* were randomly selected for qRT-PCR analysis. The results demonstrated similar gene expression trends using the qRT-PCR analysis to those obtained through the RNA-seq data (Figure 4).

### Differences in Transcript Profiles Between NaCl-Treated and Control Plants

**FIGURE 5:**
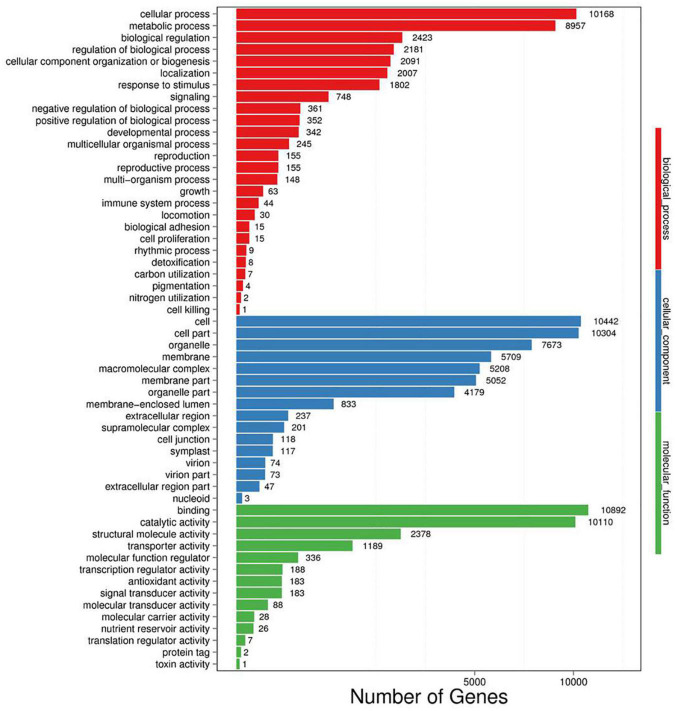
Gene ontology enrichment analysis of differentially expressed genes between 0 (control) and 150 mM NaCl- treated *Puccinellia nuttalliana*.

**FIGURE 6:**
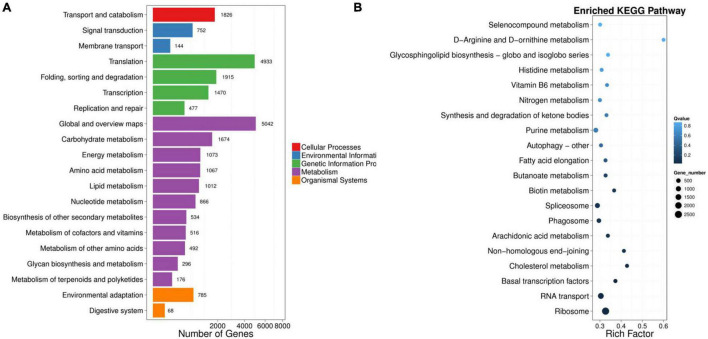
KEGG pathway classification of differentially expressed genes in roots of NaCl-treated *Puccinellia nuttalliana* plants compared to the untreated control. The five branches for KEGG pathways: cellular processes, environmental information processing, genetic information processing, metabolism, organismal systems, and drug development **(A)**, top 20 KEGG pathways of differentially expressed genes in roots of salt treated *Puccinellia nuttalliana* plants compared to the controls **(B)**. *X* axis represents enrichment factor. *Y* axis represents pathway name. The *q* value is indicated by the colors (high: white, low: blue), the lower *q* value indicates the more significant enrichment. Point size indicate DEG number (The bigger dots refer to larger amount). Rich Factor refers to the value of enrichment factor, which is the quotient of foreground value (the number of DEGs) and background value (total Gene amount). The larger the value, the more significant enrichment.

A total of 50,622 genes were differentially expressed between the NaCl-treated and control *P. nuttalliana* root samples (Figure 2D). Among these DEGs, 10,856 and 39,766 were up-and down-regulated under salt stress. We performed GO classification of the DEGs in *P. nuttalliana*. The GO classification of DEGs is shown in Figure 5. The most enriched biological process terms were cellular process, metabolic process, biological regulation, regulation of biological process, response to stimulus, and signaling. In the category of cellular components, cell, cell part, organelle, membrane, and membrane parts were highly represented. In the category of molecular functions, binding, catalytic activity, structural molecular activity, transporter activity, and transcription regulation activity were enriched. We performed KEGG pathway classification with DEGs to identify the major active biological pathways in *P. nuttalliana* in response to salt stress. The pathway classification results are shown in Figure 6A and the pathway functional enrichment results are shown in Figure 6B. The DEGs were assigned to 21 KEGG terms under five primary categories: cellular processes, environmental information processing, genetic information processing, metabolism, and organismal systems. Metabolism contained most DEGs, including carbohydrate metabolism (1,674), amino acid metabolism (1,067), energy metabolism (1,073), lipid metabolism (1,012), and nucleotide metabolism (866). Genetic information processing exhibited the second highest DEGs, followed by environmental information processing. The DEGs were subjected to the KEGG pathway enrichment analysis. Figure 6B shows the top 20 KEGG enriched pathways. The ribosome, RNA transport, arachidonic acid metabolism, fatty acid elongation, butanoate metabolism, biotin metabolism, and cholesterol metabolism categories were significantly enriched.

### Activation of Signals and Signal Transduction Pathways in Response to NaCl Stress

Multiple modifying enzymes comprising MAPKs, CIPKs, and CBLs that are involved in the signal transduction process were induced by salt stress. Increased levels of transcript abundance were found for these proteins under salt stress compared to control plants Several genes related to Ca^+2^ signaling were differentially expressed, such as calcium-transporting ATPases (*Ca^2+^ -ATPase*, Unigene24255_All) and Ca^+^/H^+^ exchangers (*CAXs*, Unigene78000_All, Unigene33268_All, and CL20809.Contig7_All) were up-regulated in response to salt stress. Increase Calcium ions signals are sensed by several calcium sensor proteins such as calcium-dependent protein kinases (*CDPK*) and calcineurin B-like (CBL) proteins. In this study, three *CBLs* (Unigene42846_All, CL2596, Contig5_All, and CL16803.Contig1_All) and 19 *CDPK* genes were up-regulated in response to salt stress. SOS3 activates SOS2 (CIPK) and forms a SOS3-SOS2 complex that triggers downstream SOS1. Under salt stress, 11 *CIPKs* were up-regulated. All differentially expressed *SOS1* (*NHE*) were down-regulated in response to NaCl stress. DEGs showed that the mitogen-activated protein kinase (MAPK) signaling pathway was perturbed due to salinity stress. MAPK cascades comprised of three kinases, MAPK, MAPK kinase (MAPKK, or MEK), and MAPKK kinase (MAPKKK, or MEKK; Joo et al., 2008). In this study, two transcripts orthologous to Arabidopsis *MAPK5* (Unigene36765_All; log2 FC = 5.23), *MAPK6* (CL2073.Contig1_All; log2 FC = 1.34) increased in abundance due to salt stress (Table 3). *MEKK* (CL288.Contig3_All; log2 FC = 6.45) and *MAPKKK3* (Unigene32660_All; log2 FC = 3.69) transcripts involved in MAPK signaling pathway were also up-regulated in response to NaCl stress (Table 3). Transcript orthologous to Arabidopsis *ACS2* (Unigene4173_All; log2 FC = −5.28) was down-regulated due to salt stress (Table 3). Other crucial signaling moieties are phospholipase C (PLC), and phospholipase D (PLD). Two PLC-like transcripts Unigene76877_All (log2 FC = 4.70) and Unigene89254_All (log2 FC = 6.18) and one PLD (Unigene80611_All; log2 FC = 5.37)-like transcripts were up-regulated in response to salt stress (Table 3).

**TABLE 3:** Fold changes of differentially expressed genes under salt stresses in the roots of *P. nuttalliana.*

Query_ID	Regulated	log2 fold change	*P*-value	Annotation
**Signal transduction pathway genes**				
Unigene24255_All	Up	5.30	9.23E-05	Calcium transporting ATPase
**CAX**				
CL17973.Contig1_All	Down	−3.75	2.29E−05	Vacuolar cation/proton exchanger CAX2.
Unigene5119_All	Down	−5.33	1.04E-06	Vacuolar cation/proton exchanger CAX2
CL17973.Contig2_All	Down	−5.08	0.0046	Vacuolar cation/proton exchanger CAX2
Unigene42846_All	Up	7.09	9.99E−06	Calcineurin B-like protein 1
CL2596.Contig5_All	Up	5.23	0.0030	Calcineurin B-like protein 4
Unigene42846_All	Up	7.09	9.99E-06	Calcineurin B-like protein 1
CL2596.Contig5_All	Up	5.21	0.0030	Calcineurin B-like protein 4
Unigene160802_All	Up	1.42	0.0026	Calcineurin B-like protein 10
CL16803.Contig1_All	Up	5.76	0.0008	CBL-interacting protein kinase 28
Unigene36765_All	Up	5.23	0.0031	MAPK5; mitogen-activated protein kinase 5
CL2073.Contig1_All	Up	1.34	0.0080	MAPK6; mitogen-activated protein kinase 6
CL288.Contig3_All	Up	6.45	8.80E-05	mitogen-activated protein kinase kinase kinase
Unigene32660_All	Up	3.69	0.0064	Mitogen-activated protein kinase kinase kinase 3
Unigene4173_All	Down	−5.29	0.0026	ACS2, 1-aminocyclopropane-1-carboxylate synthase 2
Unigene76877_All	Up	4.70	0.0106	Phospholipase C
Unigene89254_All	Up	6.18	0.0003	Phospholipase C
Unigene80611_All	Up	5.38	0.0037	Phospholipase D
Unigene20698_All	Up	5.95	0.000182	Calcium-dependent protein kinase 2
Unigene80242_All	Up	3.96	0.004923	Calcium-dependent protein kinase 12
CL19105.Contig2_All	Up	3.72	0.002681	Calcium-dependent protein kinase 7
Unigene88181_All	Up	5.70	0.002045	Calcium-dependent protein kinase 3
CL21144.Contig1_All	Up	7.46	3.02E-06	Calcium-dependent protein kinase 29
Unigene48040_All	Up	4.88	0.006771	Calcium-dependent protein kinase 2
Unigene93001_All	Up	5.09	0.006859	Calcium-dependent protein kinase 11
Unigene73511_All	Up	4.70	3.92E-07	Calcium-dependent protein kinase 29
CL21144.Contig2_All	Up	5.50	1.19E-05	Calcium-dependent protein kinase 29
Unigene24112_All	Up	5.58	0.001833	Calcium-dependent protein kinase 1;
Unigene23838_All	Up	5.57	0.000261	Calcium-dependent protein kinase 20
Unigene80411_All	Up	5.27	0.004867	Calcium-dependent protein kinase 23
Unigene139659_All	Up	4.93	0.008305	Calcium-dependent protein kinase 21
Unigene27820_All	Up	5.06	8.57E-05	Calcium-dependent protein kinase 5
Unigene165516_All	Up	3.84	0.009487	Calcium-dependent protein kinase 19
Unigene35719_All	Up	4.31	0.001686	Calcium-dependent protein kinase 29-like
Unigene53868_All	Up	4.81	0.000465	Calcium-dependent protein kinase 2
Unigene160926_All	Up	5.26	0.002987	Calcium-dependent protein kinase 15
Unigene87392_All	Up	4.95	0.006536	Calcium-dependent protein kinase SK5
**Ion transporter**				
Unigene42482_All	Up	4.13	1.41E-06	HKT8-like (HKT1;5) Na^+^ /K^+^ transporter
Unigene20014_All	Up	5.77	1.09E-05	CDK protein kinase
Unigene62798_All	Up	5.48	0.0020	Cyclin-dependent kinase CDK5
Unigene63446_All	Down	−4.41	0.0011	NHX5 Sodium/hydrogen exchanger 5
Unigene64682_All	Down	−4.50	9.54E-07	NHX6 Sodium/hydrogen exchanger 6
Unigene167080_All	Up	5.38	0.0032	Choline dehydrogenase
CL11886.Contig2_All	Up	1.84	0.0048	HAK23 Potassium transporter 23
Unigene24263_All	Up	5.61	0.0013	Potassium channel AKT1
CL14368.Contig2_All	Up	2.02	0.0035	Cyclic nucleotide-gated ion channel 1
CL11034.Contig2_All	Up	6.66	1.65E-06	Cyclic nucleotide-gated ion channel 20
CL3751.Contig3_All	Up	3.023	8.89E-05	S-type anion channel SLAH1-like
Unigene158776_All	Up	4.52	0.0043	Chloride channel
**Transcription factors**				
Unigene159865_All	Up	5.00	0.0064	Transcription factor MYB119
CL10257.Contig5_All	Up	2.88	0.0069	Transcription factor MYB59
Unigene25216_All	Up	1.41	0.0047	Transcription factor MYB12
Unigene167936_All	Up	5.09	0.0056	Transcription factor MYB3R-4
CL3152.Contig6_All	Up	4.63	5.50E-07	Transcription factor MYB37
CL8099.Contig1_All	Up	7.80	7.04E-07	bZIP9 basic leucine zipper 9
CL19018.Contig6_All	Up	4.24	0.0004	bZIP transcription factor 27
CL1946.Contig4_All	Up	1.42	0.0041	bZIP transcription factor 23
Unigene158885_All	Up	5.16	0.0063	ZIP17, HY5-like transcription factor
CL17434.Contig2_All	Down	−3.83	0.0023	WRKY transcription factor 26
CL19887.Contig2_All	Down	−4.81	0.0003	WRKY transcription factor 19
Unigene41823_All	Down	−7.87	3.24E-07	WRKY transcription factor 9
CL17890.Contig3_All	Up	2.98	0.0034	WRKY transcription factor 74
CL19032.Contig2_All	Up	1.53	0.0065	WRKY transcription factor 11
Unigene54941_All	Down	−5.27	0.0043	Ethylene-responsive transcription factor RAP2-2
CL3748.Contig7_All	Down	−5.94	0.0010	Ethylene-responsive transcription factor RAP2-3
Unigene24458_All	Up	5.12	0.0048	Ethylene-responsive transcription factor RAP2-10
Unigene12659_All	Up	7.06	1.64E-05	Nascent polypeptide-associated complex subunit alpha-like protein 3
Unigene100143_All	Up	5.24	0.0052	Nascent polypeptide-associated complex subunit alpha-like protein 3
Unigene68164_All	Up	4.80	0.0009	Nascent polypeptide-associated complex subunit alpha-like protein 1
CL18756.Contig2_All	Up	7.18	4.78E-05	NAC domain-containing protein 78
CL1250.Contig12_All	Up	4.19	0.0034	NAC domain-containing protein 13
Unigene59213_All	Down	−6.94	1.63E-r=14	Nascent polypeptide-associated complex subunit alpha-like protein 2
Unigene167020_All	Up	3.98	0.0100	ABF2-like
Unigene158994_All	Up	6.18	0.0041	DEK-domain containing protein 3, DEK3
**Aquaporins**				
Unigene159054_All	Up	7.92	2.80E-12	Aquaporin PIP2;2
Unigene76130_All	Down	−6.56	5.22E-10	Aquaporin TIP1;1
Unigene134545_All	Down	−4.99	0.0052	Aquaporin TIP2;1
Unigene19900_All	Down	−5.43	1.50E-05	Aquaporin TIP2;3
CL9482.Contig2_All	Down	−3.69	1.65E-06	Aquaporin TIP3;1
Unigene1799_All	Down	−5.72	1.04E-07	Aquaporin TIP4;1
CL11639.Contig2_All	Down	−6.94	2.12E-05	Aquaporin TIP4;2
Unigene43183_All	Down	−5.26	0.0027	Aquaporin TIP4;4
Unigene66763_All	Down	−6.39	0.0001	Aquaporin NIP1;1
Unigene71932_All	Down	−3.28	0.0014	Aquaporin NIP2;2
**Genes involved in ROS defenses**				
Unigene17356_All	Up	6.03	0.0008	Superoxide dismutase
Unigene71422_All	Up	4.34	0.0052	Superoxide dismutase
Unigene78951_All	Up	5.78	0.0010	Superoxide dismutase
Unigene43908_All	Up	4.49	0.0009	Superoxide dismutase
CL14042.Contig2_All	Up	5.37	0.0029	Superoxide dismutase
CL21164.Contig1_All	Up	5.53	0.0030	Peroxidase
Unigene45372_All	Up	4.08	0.0006	Peroxidase
Unigene49792_All	Up	1.98	0.0061	Peroxidase
CL3980.Contig1_All	Up	4.58	0.0032	Catalase
Unigene5188_All	Up	6.40	0.0002	Catalase
Unigene27478_All	Up	3.24	0.0049	Catalase
Unigene5399_All	Up	4.71	0.0096	Catalase
**Genes involved in glyceropholipids biosynthesis**				
Unigene54315_All	Down	−4.98	0.0077	Phospholipase D beta 1
Unigene67197_All	Down	−3.83	0.0014	Phospholipase D
Unigene35739_All	Down	−3.18	0.0001	N-acyl-phosphatidylethanolamine-hydrolyzing phospholipase D
CL17819.Contig2_All	Down	−3.06	0.0067	Phospholipase D1
Unigene50422_All	Down	−5.81	0.0006	Phospholipase D1
Unigene34512_All	Down	−4.48	0.0002	Phospholipase D
Unigene80611_All	Up	5.37	0.0037	Phospholipase D
Unigene29494_All	Down	−3.13	0.0043	Phospholipase D
**Genes involved in lignin biosynthesis**				
Unigene2227_All	Down	−4.09	0.0003	Caffeoyl-CoA O-methyltransferase
Unigene50175_All	Down	−4.26	4.09E-07	4-coumarate-CoA ligase 2-like
Unigene165035_All	Up	5.92	4-coumarate-CoA ligase 2	4-coumarate-CoA ligase 2
CL6032.Contig6_All	Up	1.47	0.0029	LAC17, Laccase-17
Unigene64739_All	Up	1.72	0.0046	PER39, PEROXIDASE 39
CL14735.Contig1_All	Up	5.12	0.0056	PRX34 Peroxidase 34
**Genes involved in proline biosynthesis**				
Unigene18828_All	Up	2.43	2.46E-06	P5CS2, Delta-1-pyrroline-5-carboxylate synthase 2
Unigene2907_All	Up	7.46	7.06E-06	P5CS, Delta-1-pyrroline-5-carboxylate synthase

*Functional annotation of the unigenes was performed by a BLASTx search with an *E*-value of 10-5 against seven protein databases included NR (non-redundant) protein database, non-redundant nucleotide sequence (Nt) database, GO (Gene ontology), KOG (euKaryotic Orthologous Group database), and KEGG (Kyoto Encyclopedia of Genes and Genomes protein database, SwissProt, and InterPro.*

### Ion Transporters

Transcript orthologous to *B. distachyon HKT1;5* (Unigene42482; log2 FC = 4.13) increased in abundance in salt treated plants (Table 3). Two *CDKs* genes Unigene20014 (log2 FC = 5.77) and Unigene62798 (log2 FC = 5.48), were found significantly up-regulated in response to salt stress. CDKs are a large family of serine/threonine protein kinases in regulating the cell cycle. Three transcripts orthologous to Arabidopsis *NHX5*, *NHX6*, and *NHX1* were found down-regulated in salt-treated plants. *HAK9* (CL646.Contig7_All; log2 FC = 2.90) and *HAK23* (CL11886.Contig2_All; log2 FC = 1.84) transcripts from HAK/KUP/KT family significantly increased in salt treated plants. *AKT1* (Unigene24263_All, log2 FC = 5.61), two cyclic nucleotide-gated cation channels (CNGCs; CL14368.Contig2_All and CL11034.Contig2_All) ion transporters like genes were also up-regulated under NaCl stress (Table 3). Transport of Na^+^ and K^+^ ions across the plasma membrane are driven by transport protons across the H^+^ gradient mediated by H^+^-ATPase and V-ATPase. In this study, several vacuolar ATPases and plasma membrane H^+^-transporting ATPases were up-regulated in NaCl-treated plants. Under NaCl conditions, Cl^–^ membrane transport can be conducted via the membrane transporters of multiple protein families, including CCC, cation/chloride cotransporter, and SLASH, anion channel associated homolog 1. In this study, we found that one SLASH1-like (CL3751.Contig3_All; log2 FC = 3.02) and one Cl^–^ channel-like (Unigene158776_All; log2 FC = 4.51) transcripts were up-regulated under salt stress.

### Transcription Factors and Regulators

Multiple TF families were differentially expressed in response to NaCl treatment. Here we analyzed a subset of the TF family related to salt stress. Myeloblastosis oncogene (MYB) is a large, functionally diverse protein family of which a small number are directly related to salt stress response. Unigene annotation identified 408 MYB genes of which only small members were up-regulated by salt stress. *MYB119* (Unigene159865_All; log2 FC = 5.00), *MYB59* (CL10257.Contig5_All; log2 FC = 2.88), *MYB12* (Unigene25216_All; log2 FC = 1.41), *MYB3R-4* (Unigene167936_All; log2 FC = 5.09), and *MYB37* (CL3152.Contig6_All; log2 FC = 4.63) showed highly increased transcript abundance compared to control plants. The basic leucine zipper (bZIP) represents another most diverse TF family. bZIP9 (CL8099.Contig1_All; log2 FC = 7.8) showed the highest increase in transcript abundance from this family in response to NaCl treatment. The other bZIPs that were up-regulated include *bZIP27* (CL19018.Contig6_All; log2 FC = 4.24), *bZIP23* (CL1946.Contig4; log2 FC = 1.42), and *bZIP17* (Unigene158885_All; log2 FC = 5.15; Table 3). Four *WRKY* TFs showed a decrease in transcript abundance compared to control plants under salt stress: *WRYK26* (CL17434.Contig2_All; log2 FC = −3.83), *WRYK70* (CL2822.Contig1_All, log2 FC = −2.60), *WRYK19* (CL19887.Contig2_All; log2 FC = −4.81), and *WRYK9* (Unigene41823_All; log2 FC = −7.87). Six *WRYK* genes were up-regulated, but only *WRKY39* (CL17890.Contig3_All; log2 FC = 2.99) showed greater than two log2 fold increased than control plants (Table 3). A total of 26 drebrins and related actin-binding proteins (*DREB*) were differentially expressed in response to NaCl. Among them, 15 were up-regulated with large log2 fold changes (4–7) compared to control plants. The APETALA2/ethylene-responsive element binding protein (AP2/EREBP) family TFs *RAP2.2* (Unigene54941_All) and *RAP2-3* (CL3748.Contig7_All) showed greater than five-fold (log2) downregulation, whereas *RAP2-10* (Unigene24458_All) showed greater than five-fold (log2) upregulation compared to control plants. A total of 112 NAC (NAM/ATAF1/CUC2) TFs were identified in this study. Many of the identified NAC TFs were expressed differentially in response to NaCl. *NAC3* (Unigene12659_All; log2 FC = 7.06), *NACA3* (Unigene100143_All; FC = 5.24), *NAC* (Unigene68164), *NAC107* (CL18756.Contig2_All; FC = 7.18), and *NAC48* (CL1250.Contig12_All; FC = 4.19) associated with the high upregulations of transcript levels whereas *NACA2* (Unigene59213_All; FC = −6.93) were significantly down-regulated under salt stress. Additionally, one abscisic acid-responsive (ABA) TF gene, *ABF2* (Unigene167020_All; log2 FC = 3.98), was up-regulated in response to salt stress (Table 3).

### Aquaporins

Several aquaporin genes were differentially expressed in the roots of *P. nuttalliana* as a result of the NaCl treatment. *PIP2;2* transcript (Unigene159054_All; log2 FC = 7.92) orthologus to *H. vulgare HvPIP2;2* increased in abundance in salt treated plants. Transcript abundance of the tonoplast aquaporins *TIP1;1* (Unigene76130_All; FC = −6.56), *TIP2;1* (Unigene134545_All; FC = −4.99), *TIP2;3* (Unigene19900_All; FC = −5.43), *TIP3;1* (CL9482.Contig2_All; FC = −3.69), *TIP4;1* (Unigene1799_All; FC-5.72), *TIP4;2* (CL11639.Contig2_All; FC = −6.94), and *TIP4;4* (Unigene43183_All; FC = −5.27) were significantly down-regulated by the NaCl treatment. Two nodulin-26 like intrinsic protein (NIP) genes, *NIP1;1* (Unigene66763_All; FC = −6.39), and *NIP4;2* (Unigene71932_All; FC = −3.28), were also significantly down-regulated (Table 3).

### Expression of Genes Involved in Reactive Oxygen Species Defenses

The NaCl treatment significantly induced the expression of genes involved in ROS defenses. Various enzymatic antioxidants, such as peroxidase (*POD*), superoxide dismutase (*SOD*), and catalase (*CAT*), were significantly up-regulated by NaCl (Table 3).

### Metabolite Abundance Changes in Response to NaCl Treatment

**FIGURE 7:**
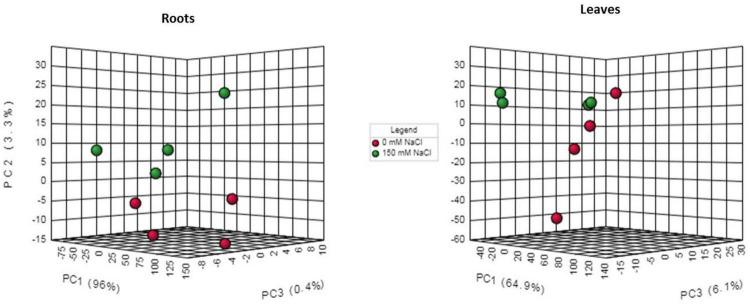
Three-dimensional principal component analysis score plot between individual samples for 0 mM NaCl and 150 mM NaCl 6-day treatments.

**FIGURE 8:**
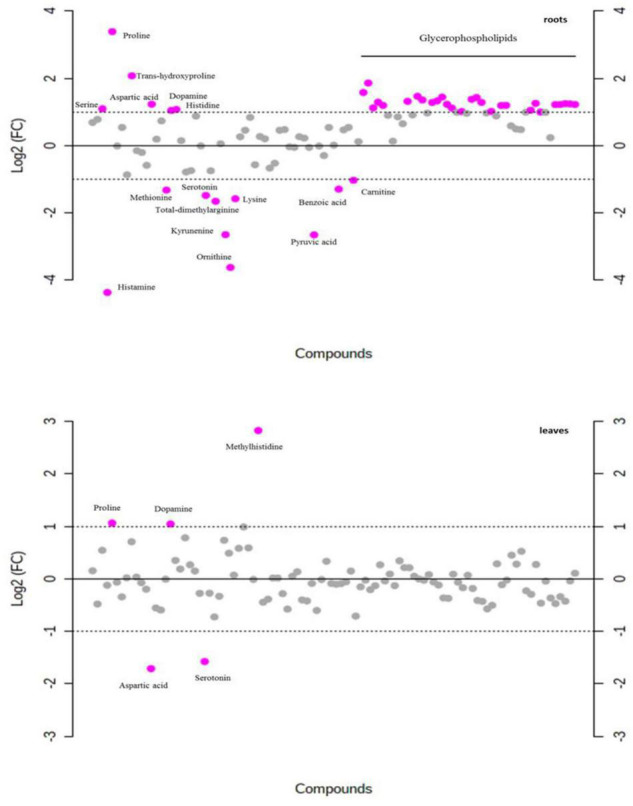
Fold-change analysis with threshold 2 of each metabolite in roots and leaves of *P. nuttalliana* treated with 150 mM NaCl for 6 days compared with the plants treated with 0 mM NaCl. The values are on log scale, so that both up-regulated and down-regulated features are plotted in a symmetrical way. Gray circles represent metabolites with no significant difference.

In total, 103 metabolites were detected in leaf and root extracts of *P. nuttalliana* by liquid chromatography-mass spectrometry (LC-MS) including different amino acids, organic acids, sugars, amines, and glycerophospholipids. The PCA showed clear separations among the control and NaCl-treated plants (Figure 7). The analysis also showed no outliers in our study and significant changes in the metabolite profiles due to the NaCl treatment. The differential abundance of metabolites evaluated by log2 fold change (Figure 8) showed that out of 103 metabolites identified in roots, 34 were up-regulated, including proline, transhydroxy proline, dopamine, histidine, serine, aspartic acid 28 glycerophospholipids. Significantly increased metabolites from the glycerophospholipids family included LYSOC16:0, LYSOC18:1, LYSOC18:2, PC36:0AA, PC36:6AA, PC aa C32:3, PC ae C34:3, PC ae C34:3, PC aa C34:4, PC ae C34:3, PC aa C34:4, PC aa C34:3, PC aa C34:2, PC aa C34:1, PC ae C36:4, PC ae C36:2, PC aa C36:5, PC aa C36:4, PC aa C36:3, PC aa C36:1, PC ae C38:4, PC ae C38:3, PC aa C38:4, PC aa C38:3, PC ae C40:3, PC ae C42:4, PC ae C42:3, PC aa C42:4, PC aa C42:2, and PC ae C44:3. In contrast, no metabolites from the glycerophospholipid family were significantly different in leaves between control and NaCl-treated plants. Only proline, dopamine, and methylhistidine concentrations were significantly increased in leaves.

### Proline and Dopamine Biosynthesis Pathways

Two transcripts of 1-delta-pyrroline-5-carboxylate synthase (*P5CS*; Unigene18828_All and Unigene2907_All) and a Pyrroline-5-carboxylate reductase (*P5CR*), the key enzyme of proline biosynthesis, were found to be up-regulated due to NaCl stress. DEGs also revealed that two ornithine d-aminotransferase transcripts were significantly up-regulated. The predicted proline biosynthesis pathway in *P. nuttalliana* is shown in Supplementary Figure 2. There are two reported dopamine synthesis pathways in plants, either via hydroxylation of tyramine or L-DOPA’s decarboxylation. A decrease in the concentration of tyrosine and an increase in the concentration of tyramine in response to NaCl treatment revealed that dopamine was produced via the hydroxylation pathway (Supplementary Figure 2).

### Metabolic Pathways Altered by NaCl Treatment

**FIGURE 9:**
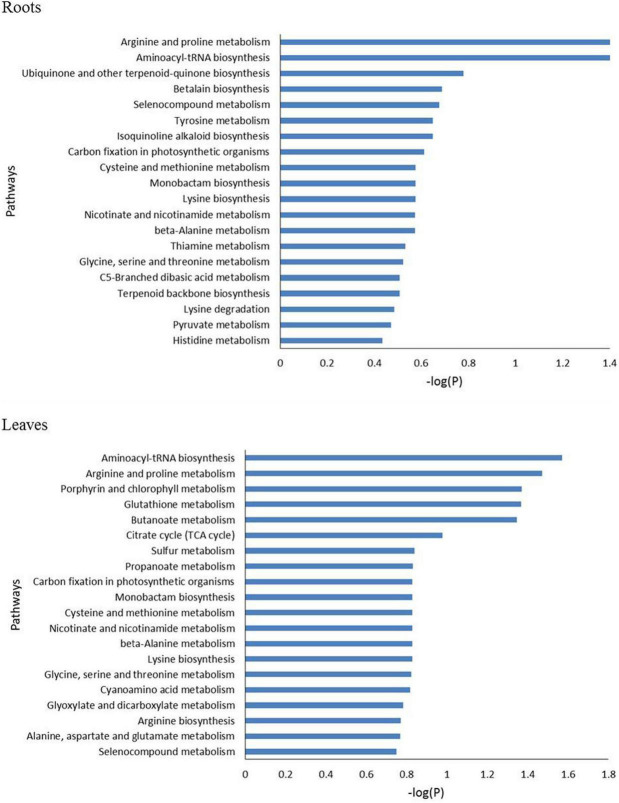
The top 20 enriched pathway analysis of significant differential accumulated metabolites in roots and leaves of *Puccinellia nuttalliana* between 0 mM and 150 mM NaCl treatments.

**FIGURE 10:**
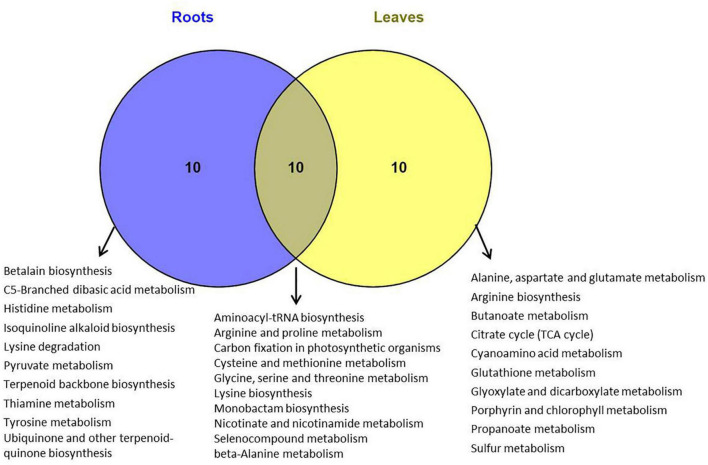
Venn diagram of KEGG pathways in which differentially metabolites were involved in roots and leaves of *Puccinellia nuttalliana* plants treated with 150 mM NaCl for 6 days.

The metabolomics data were subjected to the KEGG pathway enrichment analysis for differentially expressed metabolites induced by salt stress. The top 20 purterbed pathways in the roots and leaves of *P. nuttalliana* is shown in Figure 9. We found that 10 metabolic pathways were significantly activated in both roots and leaves (Figure 10), including arginine and proline metabolism, cysteine and methionine metabolism, glycine, serine and threonine metabolism, carbon fixation in photosynthetic organisms, seleno compound metabolism and beta-alanine metabolism. The perturbation of metabolites involved in the pathways in roots included pyruvate metabolism, histidine metabolim, betalain biosynthesis, tyrosine metabolism, ubiquinone and other terpenoid-quinone biosynthesis, lysine degradation and thiamine metabolism. In leaves, the altered pathways include the TCA cycle, butanoate metabolism, Porphyrin and chlorophyll metabolism, Alanine, aspartate and glutamate metabolism, arginine biosynthesis, glyoxylate and dicarboxylate metabolism and sulfur metabolism.

### Salt Secretion by Leaves and Cell Wall Lignification in Roots in Response to NaCl

**FIGURE 11:**
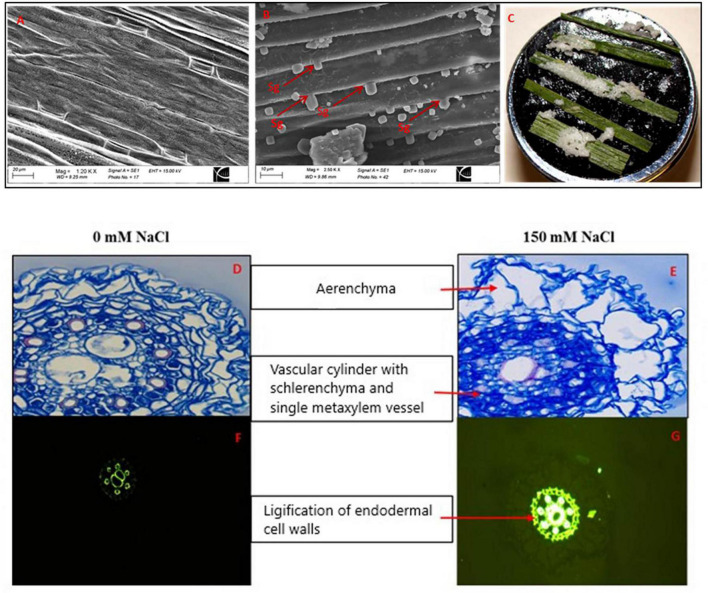
Scanning electron micrographs of the abaxial leaf surfaces from control **(A)**, 150 mM **(B)** NaCl-treated *Puccinellia nuttalliana* plants for 6 days, Sg: salt gland, leaves of *Puccinellia nuttalliana* showing salt crystals on their surfaces after the plants were treated with 150 mM NaCl treated for 6 days **(C)**, root cross sections of *P*. *nuttalliana* treated for 6 days with 0 mM NaCl **(D)** and 150 mM **(E)** NaCl; lignification of the endodermal cell walls **(F,G)** visualized by fluorescence. Cross section were made after 6 days of 0 mM **(F)** and 150 mM NaCl **(G)**.

Scanning electron microscopy (SEM) demonstrated globular materials deposited on the unwashed leaf surfaces of 150 mM NaCl-treated plants that were absent in control plants (Figures 11A,B). However, the details of salt glands were not visible in the scanning electron micrographs since they were covered by the globular materials. There were no globular materials deposited on leaf surfaces of the control plants. Salt deposition on the leaf surfaces was observed in NaCl-treated plants (Figure 11C).

Light microscopy observations revealed the presence of aerenchyma in the root cortex of *P. nuttalliana* plants. In addition, extensive sclerification in the root vascular region was observed after 6 days of 150 mM NaCl treatment and was accompanied by thickening of the endodermal cell walls. Six to eight protoxylem vessels surrounded one to two large central metaxylem vessels (Figures 11D,E). In most of the examined specimens, we observed two metaxylem vessels in control plants and one metaxylem vessel in plants treated with 150 mM NaCl (Figures 11D,E). Lignin distribution, which was examined in root sections by the fluorescence microscopy, demonstrated that the exposure to NaCl resulted in thickening of the root endodermal cell walls and the cell walls of the metaxylem vessels (Figures 11F,G). DEGs identified that caffeoyl-CoA O-methyltransferase (*CCoAOMT*), a key enzyme in the lignin biosynthesis pathway was significantly down-regulated (Unigene2227_All; FC = −4.09) in response to salt stress. We also observed that DEGs encoding *4-coumarate-CoA ligase* (Unigene165035_All; FC = 5.92), which participates in monolignol biosynthesis, was induced by the treatment. The levels of other transcripts related to lignin biosynthesis, including *LAC17* (CL6032.Contig6_All), *PER39* (Unigene64739_All), *PRX34* (CL14735.Contig1_All), also increased in plants treated with NaCl (Table 3).

## Discussion

In the present study, we performed *de novo* transcriptome sequencing and metabolomic analyses of *P. nuttalliana* plants treated in hydroponic culture with 0 and 150 mM NaCl to gain better insight into the salt tolerance mechanisms. We were especially interested in finding clues that would help explain the ability of plants to maintain water balance through the earlier reported enhancement of cell hydraulic conductivity in the roots of *P. nuttalliana* exposed to NaCl (Vaziriyeganeh et al., 2018). Appropriate salt concentrations are required for optimal growth of true halophytes (Yuan et al., 2019). For example, the optimal NaCl concentration for the growth of *Suaeda salsa* was 200 mM (Yang et al., 2010) while 100 mM NaCl was found to be optimal for *Cakile maritima* (Debez et al., 2004). A previous study showed that the NaCl treatments for 10 days with the concentrations as high as 150 mM enhance shoot and root growth of *P. nuttalliana* while no growth enhancement was observed in plants treated with 300 mM NaCl (Vaziriyeganeh et al., 2018). In the present study, we successfully identified 242,833 unigenes with an average length of 742 bp. A high percentage of *P. nuttalliana* sequences closely matched sequences of *A. tauschii*, which tolerates salinity through an effective Na^+^ homeostasis strategy (Wei et al., 2008; Abbas et al., 2021). Furthermore, we obtained a set of SSR markers from the RNA-Seq data of the roots of *P. nuttalliana.* The development of large number of SSRs in the *P. nuttalliana* will be useful in the marker-assisted breeding and ecological studies.

### Aquaporins Are Involved in Salt Tolerance in *P. nuttalliana*

In our earlier study (Vaziriyeganeh et al., 2018), we demonstrated that net photosynthesis and transpiration rates in *P. nuttalliana* were not affected by the treatments of up to 150 mM NaCl. Interestingly, the cell hydraulic conductivity in roots was enhanced when measured in plants treated for 3 days with 50 and 150 mM NaCl (Vaziriyeganeh et al., 2018). A subsequent study demonstrated that the aquaporin-mediated transport was enhanced by Na^+^ since similar effects were observed in plants treated for 6 days with 150 mM NaCl and 150 mM Na_2_SO_4_, but not with 150 mM KCl (unpublished). In all studied species of glycophytic plants, cell hydraulic conductivity and aquaporin-mediated root water transport were inhibited by NaCl (Martinez-Ballesta et al., 2003; Boursiac et al., 2005; Sutka et al., 2011; Vaziriyeganeh et al., 2018). The NaCl concentration that inhibited cell hydraulic conductivity in roots of *Arabidopsis* was as low as 10 mM (Lee and Zwiazek, 2015). Maintenance of water balance in plants exposed to salinity requires a coordinated effort that may involve a combination of salt exclusion and salt tolerance processes to minimize direct ion toxicity and osmotic effects of salt. Contrary to TIPs, NIPs, and to most of the PIP aquaporins, NaCl triggered a significant increase in root *PIP2;2* transcripts in *P. nuttalliana*, which could contribute to the earlier observed increase in root cell hydraulic conductivity by NaCl (Vaziriyeganeh et al., 2018). It cannot be excluded that PIP2;2 may also play a dual role under salt stress by facilitating Na^+^ translocation since some of the aquaporins were demonstrated to function as ion transporters. Studies in *Arabidopsis* demonstrated that PIP2;1 and PIP2;2 function as water and ion channels in heterologous systems suggesting their role as non-selective cation channels (NSCC) responsible for Na^+^ entry into plant roots (Byrt et al., 2017; McGaughey et al., 2018). However, the possibility that PIP2;2 may also act as an ion transporter needs to be further examined. It also remains unclear how the function of PIP2;2 can be maintained in *P. nuttalliana* in the presence of Na^+^ and what specific properties of the PIP2;2 protein make it more desirable under NaCl stress compared with the other PIPs, which had their transcripts down-regulated by the NaCl treatment. Transcriptome profiling of a halophytic plant *Kochia sieversiana* revealed that 10–12 aquaporin genes were up-regulated by mild salinity while only two aquaporins were up-regulated by high salinity and it was suggested that these aquaporins contribute to salinity and osmotic tolerance (Zhao et al., 2017). However, the authors did not specify which aquaporins were up-regulated by salt stress. Downregulation of several *TIPs* in *P. nuttalliana* by the NaCl treatment may also have important consequences to water homeostasis by regulating water fluxes across the tonoplast. Several studies revealed that TIP aquaporins, which are primarily located in the vacuolar membrane (Maurel et al., 2008), play important roles under salt stress (Afzal et al., 2016; Liu X. et al., 2020). In addition to the intracellular water movement, TIPs are involved in transporting small solutes and gases (Nozaki et al., 2008). A study with a succulent halophyte *Mesembryanthemum crystallinum* also showed that the transcript abundance of *TIP1;2* decreased in response to salt stress, suggesting that TIPs may play a role in salt responses of halophytes (Kirch et al., 2000). On the other hand, higher expression levels of both *AoPIPs* and *AoTIPs* were observed in the shoots of the mangrove tree *Avicennia officinalis* treated with 500 mM NaCl (Tan et al., 2013). The increased expression levels of *TIPs* by NaCl in *A. officinalis* and the decreased expression of *TIPs* in P. *nuttalliana* are likely related to the differences in plant salt resistance strategies of these two halophytes.

It is also noteworthy that two NIPs were up-regulated in *P. nuttalliana* by salt, pointing to their possible roles in the salt tolerance mechanisms. NIP2 homolog aquaporin was identified as a Si transporter (Ma and Yamaji, 2006). Under salinity stress, Si uptake by the NIP aquaporins may reduce Na^+^ conglomeration in cell membranes and improve water transport (Rios et al., 2017). Overexpression of a wheat NIP aquaporin (TaNIP) in Arabidopsis was also demonstrated to improve plant salt stress tolerance, which was associated with an increase in K^+^/Na^+^ ratios in roots of salt-stressed overexpression plants compared with the wild-type plants (Gao et al., 2010).

### Osmoprotectants in Salt Tolerance of *P. nuttalliana*

Our metabolomic analysis demonstrated that proline accumulation is among salt stress tolerance mechanisms in *P. nuttalliana*. We found a significant increase in leaf and root proline concentrations after 6 days of the NaCl treatment. Our RNAseq data suggested that the glutamate and ornithine pathways contributed to proline biosynthesis in *P. nuttalliana*. The significance of the glutamate and the ornithine pathways in proline biosynthesis in salt-treated plants is not clear. However, proline is synthesized mainly from glutamate and plants use ornithine as an alternative pathway for proline biosynthesis (Szabados and Savoure, 2010; An et al., 2013). Our results show that *PROT2* expression was up-regulated in *P. nuttalliana* roots by the NaCl treatment. PROT2 is presumed to be involved in xylem-to-phloem transfer and long-distance transport of proline (Zhang et al., 2010; Yao et al., 2020). We found the concentrations of free amino acids to be higher in leaves compared with roots under both control and salt stress conditions. Free amino acids are used for osmotic adjustment in numerous plant species (Jiménez-Arias et al., 2021). Several amino acids transporters may be involved in the distributions of amino acids in different plant parts (Yao et al., 2020). Two members of the amino acid permease (AAP) family, *AAP3* and *AAP7* were up-regulated in *P. nuttalliana* in response to NaCl treatment indicating their involvement in the redistribution of root-derived amino acids to leaves. AAP3 is preferentially expressed in root phloem and localized in the endodermal cells in root tips (Okumoto et al., 2004).

We identified *P. nuttalliana* as a dopamine- (3-hydroxytyramine or 3,4-dihydroxyphenethylamine) and serotonin-rich plant species, which likely contributes to its ability to withstand high salt concentrations. In mammals, dopamine and serotonin have been characterized as a neurotransmitters. The absence or deficiency of dopamine in humans can cause Parkinson’s disease, depression, and impaired motor control (Belujon and Grace, 2017). In plants, dopamine contributes to abiotic stress tolerance, including drought and salt stress, by affecting the expression of many stress-related genes (Liu Q. et al., 2020). Dopamine is a strong antioxidant (Kulma and Szopa, 2007) and it can reduce oxidative damage through direct scavenging of oxidants or enhance antioxidative enzyme activities in plants affected by salt (Li et al., 2015). Dopamine was also suggested to alleviate salt−induced stress by regulating SOS pathway (Li et al., 2015) through its effect on the expression of Na^+^/H^+^ antiporters including SOS1 (Gao et al., 2020; Marchiosi et al., 2020). Dopamine may also play a role in regulating water transport in plants affected by salinity. In rice plants, the *OsPIP1-3* transcript was up-regulated by salt stress, whereas exogenous application of dopamine decreased the *OsPIP1-3* expression under salt stress (Abdelkader et al., 2012). There are two reported dopamine production pathways in plants, either via hydroxylation of tyramine or decarboxylation of L-DOPA. In *P. nuttalliana*, tyrosine decarboxylase’s upregulation and decreased concentration of tyrosine increased concentration of tyramine under salt treatment revealing that dopamine is synthesized via hydroxylation of tyramine. Understanding the underlying molecular mechanisms of dopamine biosynthesis is vital for stabilizing the metabolic processes by modifying the dopamine metabolic pathway to improve salt tolerance in glycophytes. Serotonin was identified in both leaves and roots of *P. nuttalliana* and was significantly down-regulated in roots by the NaCl treatment, but not in leaves. Serotonin is a neurotransmitter in mammals (Belujon and Grace, 2017). In plants, serotonin may serve multiple functions that have not been well characterized. It is known to be involved in plant growth and development processes, as well as in responses to biotic and abiotic stresses (Erland et al., 2016). A recent study showed that an application of exogenous serotonin increased leaf chlorophyll concentrations in salt-stressed rapeseed plants and activated antioxidant enzyme systems including CAT, SOD, and POD (Liu et al., 2021).

### Signal Transduction and Activation of Salt Signaling Pathways

Salt stress resistance of halophytes is modulated by multiple sensors and signaling pathways, including Ca^2+^, SOS pathway and MAPK cascades, ROS, and hormones (Yang and Guo, 2018; Isayenkov and Maathuis, 2019) Ca^2+^ accumulation in cells can be triggered through the PLC mechanism (Putney and Tomita, 2012). In *P. nuttalliana*, two PLC and one PLD transcripts were up-regulated in response to NaCl stress. PLD and phosphoinositide-specific PLC pathways are involved in the production of phosphatidic acid (PA), a signaling lipid, which rapidly increases in response to drought and salinity stresses (Mueller-Roeber and Pical, 2002; Hong et al., 2010; McLoughlin and Testerink, 2013). PA interacts with MPK6 and activates the phosphorylation of SOS1 (Yu et al., 2010; Yang and Guo, 2018). The increase in Ca^2+^ in the cytoplasm in response to salt stress results in the activation of CBL/CIPKs in the SOS signaling pathway to control Na^+^ transport in halophytes (Nikalje et al., 2017). In *P. nuttalliana*, several SOS pathway genes, *CBLs* and *CIPKs* were differentially expressed in response to NaCl. *CBL1*, *CBL3*, *CBL10*, *CIPK32*, *CIPK26*, *CIPK18*, *CIPK8*, *CIPK11*, *CIPK21*, *CIPK23*, *CIPK28 CIPK1*, and *CIPK9* were up-regulated, which demonstrated that SOS was active in response to NaCl. CBL1 and CBL10 were identified as positive regulators of salt stress in *Arabidopsis* plants (Quan et al., 2007), and OsCPK21 was demonstrated to improve salt stress tolerance in rice (Asano et al., 2011). We identified multiple transcripts of the three MAPK kinases, which participate in the mitogen-activated protein kinase pathway and were upregulated by NaCl, including *MAPKKK*, *MAPKK*, and *MAPK*. A *MPK6* transcript that was up-regulated in this study was reported to be involved in ethylene biosynthesis via phosphorylation of ACS2/ACS6 (Liu and Zhang, 2004).

### Reactive Oxygen Species Scavengers

Salt stress triggers excessive ROS formation resulting in oxidative stress. Plants have effective non-enzymatic and enzymatic antioxidant defense systems for scavenging ROS including SOD, CAT, and POD. SOD converts the superoxide radical into the less toxic hydrogen peroxide. H_2_O_2_ is then scavenged by POD, CAT or other scavenging enzymes (Nikalje et al., 2018; Xiong et al., 2019). In our study, five *Cu, Zn-SODs*, five *Fe-SOD*, four *Mn-SODs*, four *CATs*, and six *PODs* were up-regulated by NaCl. These enzymes likely contributed to salt tolerance of *P. nuttalliana* by alleviating oxidative damage via ROS scavenging activity.

### Ion Homeostasis

Similarly to other studies in salt tolerant plants (Cuin et al., 2008; Reddy et al., 2017), and our earlier study with P. *nuttalliana* (Vaziriyeganeh et al., 2018), we measured high K^+^/Na^+^ ratios in plants subjected to the 150 mM NaCl treatment. The high K^+^/Na^+^ ratio indicates low Na^+^ selectivity in the presence of NaCl, which is important for plants to survive in saline environments. Several ion transporters are known to control K^+^/Na^+^ ratio. The high-affinity K^+^ transporter (HKT) protein family protects plants by controlling excess Na^+^. In rice, OsHKT1;5 was found to be involved in Na^+^ exclusion from xylem to regulate K^+^/Na^+^ homeostasis during salt stress (Kobayashi et al., 2017). *Thellungiella halophila* and *Puccinellia tenuiflora* halophytic plants exhibit strong selectivity for K^+^ over Na^+^ via HKT proteins (Taji et al., 2004; Wang et al., 2004; Ren et al., 2005; Volkov and Amtmann, 2006). In the present study, DEGs analysis of RNAseq data revealed that *HKT1;5* was significantly up-regulated in *P. nuttalliana* by NaCl. Based on our finding, we hypothesized that *P. nuttalliana* rely on HKT1;5 to maintain balance between Na^+^ and K^+^ in the cytoplasm upon salt stress by controlling the Na^+^ efflux and distribution into various organs. We also found that several antiporters including *HAK9*, *HAK23*, *AKT1*, and *AKT3* were induced by NaCl in *P. nuttalliana*. These genes might function as an effective pathway for K^+^ and Na^+^ uptake under NaCl stress. Several *CNGCs* (The cyclic nucleotide-gated channels) were significantly up-regulated by salt stress. It is possible that these genes in addition to participating in K^+^ and Na^+^ acquisition might be involved in Na^+^ loading into the xylem to maintain the ionic balance when exposed to NaCl. Plasma membrane Na^+^/H^+^ antiporter SOS1 and vacuolar Na^+^/H^+^ antiporters NHX1 help maintain high K^+^/Na^+^ ratio in the cytosol and sequestration of Na^+^ (and Cl^–^) in the vacuoles, a key salt resistance features of halophytes (Nikalje et al., 2017). Surprisingly, all vacuolar *NHXs* were down-regulated due to NaCl stress in *P. nuttalliana*. This indicates that these genes in *P. nuttalliana* may have different function or the stress triggered the upregulation of *NHX1* transcript to increase active accumulation of K^+^ in the vacuoles to reduce the Na^+^ sequestering into the vacuoles and enhance Na^+^ loading into the xylem of roots by other antiporters. Halophytes use Na^+^ as an energy-efficient source of osmolytes to maintain their maximum growth under saline conditions (Nikalje et al., 2017). The stress that is induced in salt-loving plants by the lack of adequate salt in the root medium triggers plant responses that obscure the mechanisms involved in their salt resistance. Another possibility is that *P. nuttalliana* does not depend on NHX to compartmentalize Na^+^ and K^+^ and, instead, relies on the synthesis of compatible organic solutes (Yokoi et al., 2002). We also found that all of the differentially expressed *SOS1* genes were down-regulated in control plants (0 mM NaCl) compared with NaCl-treated plants. In a salt-free environment, the plants likely suffered from stress that led to the upregulation of *SOS1* genes to transport more Na^+^ from roots to shoots through the xylem. In *T. halophila*, *SOS1* transcript was also more strongly induced in control plants compared with salt-treated plants (Kant et al., 2006). The authors suggested that SOS1 is involved in xylem loading of Na^+^ under control conditions. Similarly, transcriptome profiling of a halophytic plant *K. sieversiana* showed that the expression of *NHX* genes was not induced by salinity stress (Zhao et al., 2017). The authors suggested that the genes known to be involved in salinity tolerance in glycophytic plants may play different roles and may not be involved in salinity tolerance mechanisms of *K. sieversiana.*

The concentration of Cl^–^ was significantly higher in shoots compared with roots of *P. nuttalliana* treated with NaCl. Halophytic plants can accumulate Cl^–^ in shoot tissues in concentrations as high as 1.5M (Bazihizina et al., 2019). Accumulation of Cl^–^ is less energy demanding compared with its exclusion and halophytes require high concentrations of Cl^–^ for optimal photosynthetic activity (Bazihizina et al., 2019).

### Activation of Salt-Responsive Transcription Factors Regulating Gene Responses to NaCl

Transcription factors play an essential role in plant stress tolerance by regulating the transcription of the downstream genes via binding to a cis-regulatory specific sequence of the target genes. Various families of TFs such as AP2/ERF, bZIP, MYB, WRKY, and NAC, with the links to salt tolerance (Nikalje et al., 2017) were differentially expressed in this study. Four WRKY TFs (*WRKY11*, *WRKY57*, *WRKY42*, and *WRKY39*) were significantly up-regulated in *P. nuttalliana* by the NaCl treatment. It has been reported that WRKY11 responds to various environmental stresses. Overexpression of alfalfa WRKY11 improved salt tolerance in soybean (Wang Y. et al., 2018). The chromatin-associated protein DEK3, up-regulated in *P. nuttalliana* in response to NaCl, was identified as a salt-tolerant protein in the earlier studies (Waidmann et al., 2014). Several *bZIP* genes were differentially expressed in response to the NaCl treatment. In transcriptome profiling of the halophytic turf grass *Sporobolus virginicus*, five *bZIP* genes were differentially expressed in roots in response to salt stress (Yamamoto et al., 2015). The Long Hypocotyl 5 (*HY5*) TF, that was up-regulated by NaCl in *P. nuttalliana*, was reported to regulate several stress-responsive genes, including *MYB59* and *DREB2A* (Maxwell et al., 2003; Du et al., 2019; Skalak et al., 2021). These genes were also up-regulated in our study in *P. nuttalliana*. ABF2 functions as a positive regulator of the ABA signaling pathway in drought and salt stresses (Wu et al., 2020). A previous report on the halophyte *Salicornia persica* transcriptome profiling identified that the *ABF2* gene was up-regulated under salinity conditions (Aliakbari et al., 2021). Our study found that an *ABF2*-like transcript was up-regulated by NaCl, indicating that ABF2 plays a regulatory role in ABA signaling *in P. nuttalliana*.

### Pathways Involved in Salt Tolerance Response in *P. nuttalliana*

We explored the pathways linked to salinity tolerance based on differential metabolites. In leaves, the salt-induced metabolites were associated with the TCA cycle, butanoate metabolism, as well as porphyrin and chlorophyll metabolism. Butanoate metabolism is closely associated with the PA pathway involved in salt stress signaling (Yu et al., 2010; Zhang et al., 2014). Butanoate metabolism pathway was also identified in salinity responses of salt tolerant wheat (Xiong et al., 2017). TCA cycle intermediates including citric acid, succinate, pyruvate, and 2-oxoglutarate significantly increased in leaves. Since TCA cycle is essential for energy production and maintaining various physiological processes (Fernie et al., 2004), these increases point to a boost of ATP production in *P. nuttalliana* by the NaCl treatment. Several pathways were preferably enriched with differential metabolites in roots, including pyruvate, histidine, thiamine and tyrosine metabolism. In both roots and leaves, salt-induced pathways included glycine, serine and threonine metabolism, arginine and proline metabolism, cysteine and methionine metabolism and beta-alanine metabolism. Although some of these alterations of the amino acid metabolism pathways may be directly linked to the salt-tolerance mechanisms, more studies are required to clarify their significance in salt responses of this halophytic plant.

### Involvement of Glycerophospholipids in Salt Tolerance Responses in *P. nuttalliana*

Salt tolerance of *P. nuttalliana* likely involves the alterations in the membrane lipids as evidenced by a significant increase in the metabolites of the glycerophospholipid family in roots. In addition to their functional role as structural constituents of membranes, glycerophospholipids are involved in the plant hormone signal transduction (Bruntz et al., 2014). Several *PLD*-like trancripts were repressed by salt stress in *P. nuttalliana*. PLD regulates the intercellular signaling and metabolic pathways under stress conditions (Bruntz et al., 2014). It hydrolyses the phosphodiester bond of the glycerolipid phosphatidylcholine to form PA and choline (Brown et al., 2017). PLD and PA play significant roles in plants under drought and salinity stress (Hong et al., 2010).

### Adaptations of Plant Structure to Salinity

The SEM demonstrated globular materials covering the salt glands on unwashed leaf surfaces. Salt glands are commonly present in halophytic plants (Oi et al., 2013). Light microscopy observations of *P. nuttalliana* root sections also revealed increased cell wall lignification of the epidermis and root metaxylem vessels of the NaCl-treated plants. Lignin biosynthesis pathway includes the monolignol biosynthesis step. The 4-coumarate-CoA ligase enzyme catalyzes the first step to provide precursors for downstream metabolites (Fraser and Chapple, 2011) and was differentially expressed in the present study. A *CCoAOMT* transcript, which works downstream of *4CL* (Xie et al., 2018) was significantly downregulated in the present study. Downregulation of *CCoAOMT* gene was predicted to increase wood density in *Populus trichocarpa* by affecting lignin biosynthesis (Wang J. P. et al., 2018). Enhanced cell wall lignification in root cells is considered to be the salt tolerance feature in halophytes (Barzegargolchini et al., 2017). In addition, root aerenchyma structures that were formed in *P. nuttalliana* in response to NaCl may have an important role in improving root aeration under stress. Increased formation of aerenchyma was reported for maize (Zhu et al., 2010) and rice (Karahara et al., 2012) roots under osmotic stress conditions. Root sclerification, which was observed in *P. nuttalliana* has been commonly reported for plants exposed to salinity and other environmental stresses and plays a key role in regulating nutrient uptake and radial water flow in roots (Lux et al., 2004). This feature together with the thickening of endodermal cell walls in *P. nuttalliana* following salt treatment may serve as a cellular barrier reducing salt uptake into the stele through apoplastic pathway under salinity conditions (Chen et al., 2011).

## Conclusion

**FIGURE 12:**
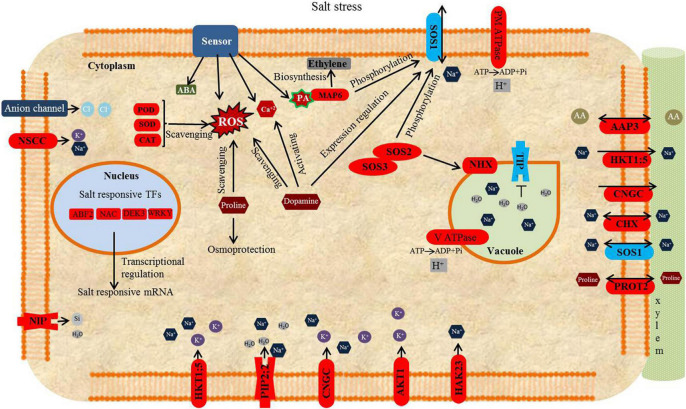
A model proposed for the responses of *Puccinellia nuttalliana* to NaCl based on root transcriptomic and metabolomic analyses. NaCl is sensed by the salt sensors which triggers cytosolic Ca^2+^ accumulation in the cytosol. Ca^2+^ activates SOS3 leading to the formation of SOS3-SOS2 complex. This complex triggers the activation of downstream SOS1. Salt stress induces the activation of NaCl-responsive TFs, important regulators of salt responsive genes. TIPs are down-regulated to slow down water efflux from vacuoles. PIP2;2 is up-regulated to lower resistance of plasma membrane to water influx into the cytoplasm and transport of Na^+^ ions. Several antiporters, NHX, HKT, SOS1, PIP2;2, AKT, HAK, CNGG, and NSCC are differentially expressed to facilitate intra- and intercellular K^+^ and Na^+^ homeostasis in *Puccinellia nuttalliana* under salt stress. Na^+^ transport across the plasma membrane driven by the H^+^ gradient occurred by plant proton pumps, H^+^-ATPase and V-ATPase. Na^+^ is sensed by sensors leading to the enhanced accumulation of ROS. The function of proline accumulation under salt stress is to provide osmotic adjustment and scavenging of ROS. Dopamine improves the antioxidant capacity by scavenging ROS, regulate the expression of SOS1 and activating Ca^2+^ signaling pathway. Phosphatidychlines increased for membrane lipid remodeling to regulate fluid and ion permeability and maintain membrane integrity under saline environment. Salt stress elevates the level of PA, a direct activator of MPK6. MPK6 e is involved in regulating ethylene biosynthesis and phosphorylation of SOS1. Amino Acid Permease3 (AAP3) plays a potential role in the uptake and distribution of amino acids. L-Proline transport through plasma membrane is achieved L-proline transporter (ProT2). SOS—salt overly sensitive, PIP-Plasma membrane intrinsic protein, NHX—sodium/proton antiporters, PM-ATPase—plasma membrane ATPase, V-ATPase—vacuolar H ATPase, V-PPase—vacuolar pyrophosphatase, HKT—high affinity potassium transporter, HAK—high affinity K^+^ transporter, AKT—Arabidopsis K^+^ Transporter, ROS—reactive oxygen species, ATP—adenosine tryphosphate, ADP—adenosine diphosphate, TF—transcription factor. CNGC—cyclic nucleotide-gated cation channel; NSCC—non-selective cationic channel, PIP—plasma membrane intrinsic protein, ROS—reactive oxygen species. PA—phosphatidic acid, ABA—Abscisic acid. Phosphatidychlines increased to for membrane lipid remodeling to regulate fluid and ions permeability and maintain membrane integrity. Transcript upregulation denoted by red and downregulation by blue.

The present study provided a comprehensive overview of transcriptomic and metabolomic changes in *P. nuttalliana* exposed to 0 and 150 mM NaCl. The study revealed important salt tolerance strategies that shed new light on the complex processes contributing to the maintenance of water balance in this halophytic plant. We propose a model (Figure 12) for salt tolerance mechanisms in *P. nuttalliana* based on the DEGs and deregulated metabolites as well as structural modifications. We identified several ion transporters, including HKT1;5 and PIP2;2 that likely play major roles in ion homeostasis, water balance, and water transport under salt stress. The study also identified *P. nuttalliana* as a dopamine-rich plants and NaCl induced an additional increase of dopamine concentrations in the root tissues. Overall, both metabolomic and transcriptomic analyses revealed that *P. nuttalliana* responds to salt stress by complex alterations in the nucleotide and amino acid metabolism, TCA cycle, and porphyrin and chlorophyll metabolism. Further studies of these genes and metabolites will provide additional insight into their functional significance.

## Data Availability Statement

The datasets presented in this study can be found in online repositories. The names of the repository/repositories and accession number(s) can be found below: https://www.ncbi.nlm.nih.gov; PRJNA631059.

## Author Contributions

MV performed all experiments, analyzed data, and wrote the manuscript. SK helped with RNA-seq and metabolomics data analyses and interpretations, and writing the manuscript. JZ developed the concept and was involved in aspects of the experiments and manuscript writing. All authors contributed to the article and approved the submitted version.

## Conflict of Interest

The authors declare that the research was conducted in the absence of any commercial or financial relationships that could be construed as a potential conflict of interest.

## Publisher’s Note

All claims expressed in this article are solely those of the authors and do not necessarily represent those of their affiliated organizations, or those of the publisher, the editors and the reviewers. Any product that may be evaluated in this article, or claim that may be made by its manufacturer, is not guaranteed or endorsed by the publisher.

## Footnotes

1
https://primer3.ut.ee/

2
https://imagej.nih.gov/ij/

## Supplementary Material

Supplementary Figure 1 Functional distribution of GO annotation.

Supplementary Figure 2 Proline and dopamine biosynthesis pathways identified in *P. nuttalliana.*

Supplementary Table 1 Clean reads quality metrics.

Supplementary Table 2 Primer list used for qRT-PCR valiodation of RNAseq data in *Puccinellia nuttalliana* to salt stress.

Supplementary Table 3 Quality metrics of Unigenes.

Supplementary Data Sheet 1 Polymorphic SSRs primer pairs derived from *Puccinellia nuttalliana* unigenes.
