# Supplementary material for: Transcriptome and Metabolome Analyses Reveal Potential Salt Tolerance Mechanisms Contributing to Maintenance of Water Balance by the Halophytic Grass Puccinellia nuttalliana
Source: Front Plant Sci. 2021 Oct 29;12:760863. doi: 10.3389/fpls.2021.760863 (PMC8586710; doi:10.3389/fpls.2021.760863)
Supplement: Supplementary Figure 1 — Functional distribution of GO annotation. [file Data_Sheet_1.docx]

Supplementary Material

#
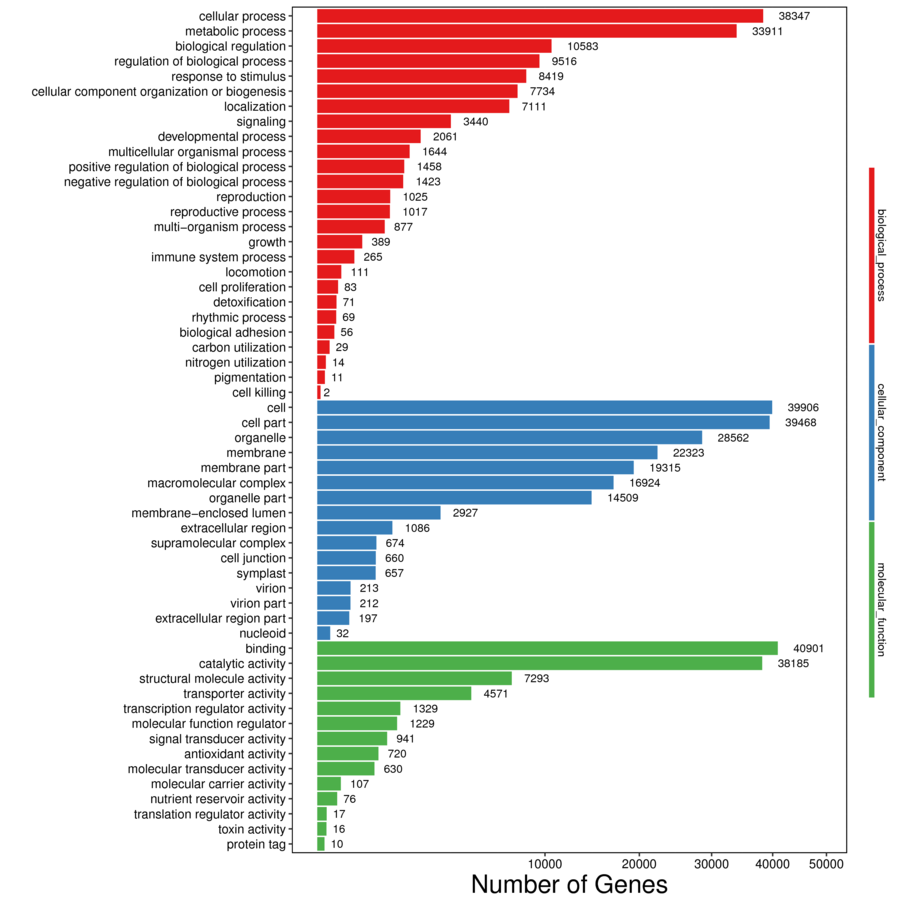


# Supplementary Figure S1. Functional distribution of GO annotation.


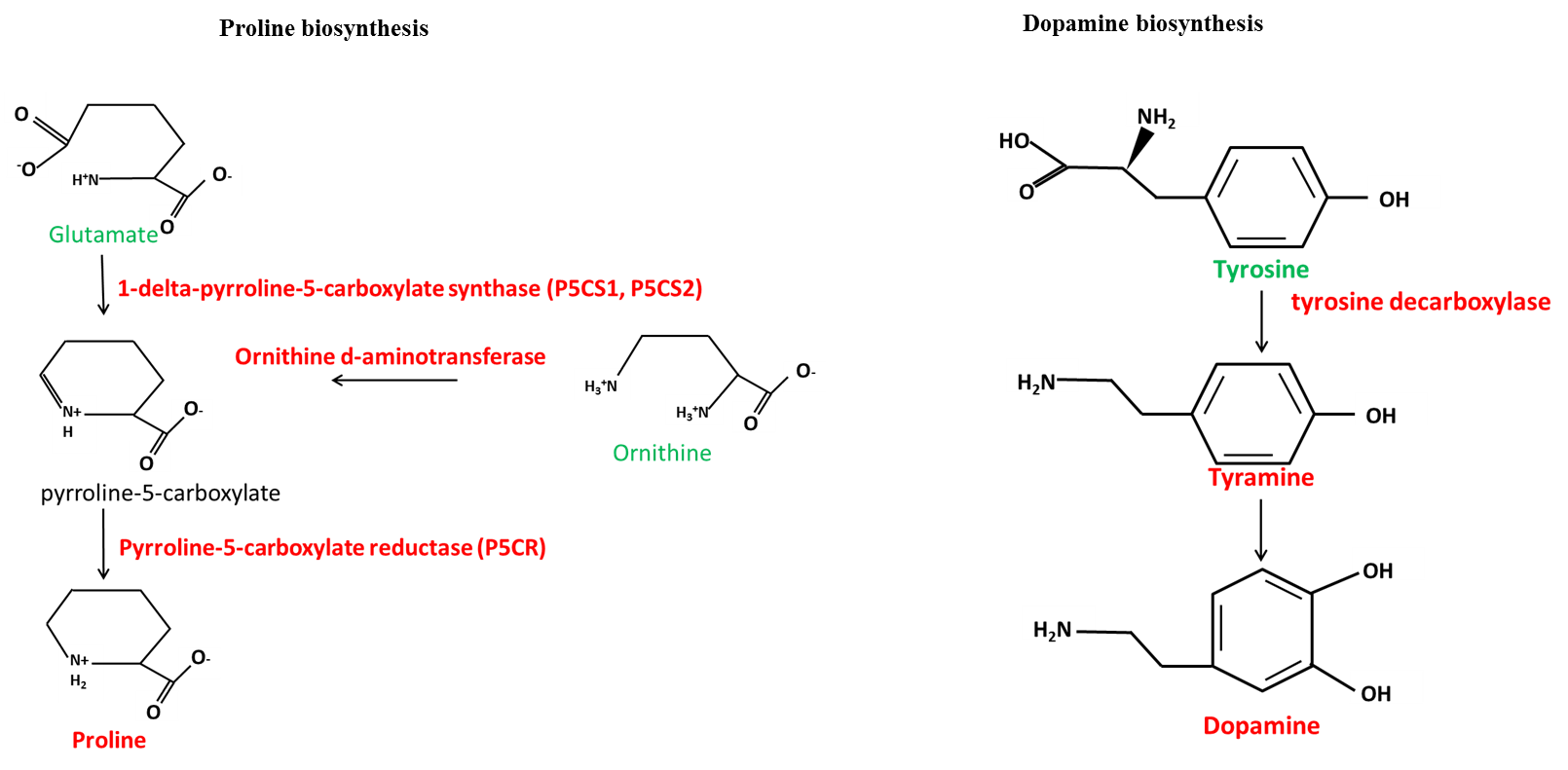


**Supplementary Figure S2.** Proline and dopamine biosynthesis pathways identified in Puccinellia nuttalliana. Proline is synthesized via two pathways mainly from glutamate and alternatively ornithine. The enzymes carrying out the reactions shown in the figure are as follows: pyrroline-5-carboxylate synthase (P5CS1, P5CS2), ornithine aminotransferase (OAT); pyrroline-5-carboxylate reductase (P5CR). Red colour denoted increased and green colour denoted decreased. The decarboxylation of tyrosine by tyrosine decarboxylase (TYDC) produces tyramine then converted to dopamine.

**Supplementary Table S1.** Clean reads quality metrics.

**Supplementary Table S2**: Primer list used for qRT-PCR validation of RNAseq data *in Puccinellia nuttalliana* to salt stress. Primers efficiency, correlation coefficient (R^2^) and slope of standard curves for the genes analyzed in this study.

| Primer ID | Primer sequence | R^2^ | Slope | Effciency (%) |
| --- | --- | --- | --- | --- |
| *PIP2;2* F | CGTCAGCTGCTACAGTTCTT | 0.98 | -3.51 | 92.71 |
| *PIP2;2* R | GACGATCACATCGAGACCATATT |  |  |  |
| *HKT1;5* F | CACCTACGTCAAGTCCAAGAAG | 0.96 | -3.29 | 101.35 |
| *HKT1;5* R | CAGGGTCGTGCATCATAGATATAG |  |  |  |
| *TIP1* F | CACCACCGACTACTAAGCTAAAG | 0.98 | -3.25 | 103.09 |
| *TIP1* R | GGTTCATGGAACGGGAAGAA |  |  |  |
| *DUF* F | GTCCCGTTACAATGTGGTAGAT | 0.97 | -3.13 | 108.68 |
| *DUF* R | GGTCGATCCTAGAGTCCTGAT |  |  |  |
| *HAK9 F* | CGGCACGGCGTTCATAAT | 0.98 | -3.29 | 101.34 |
| *HAK9 R* | GGCGCAGGAAGTTGTACC |  |  |  |
| *WRKY17* F | CGGGCATGCCTAACAACTTA | 0.95 | -3.19 | 105.82 |
| *WRKY17* R | CCTTCTTTACACGGAACAACATTC |  |  |  |
| *CBL10* F | TGCAAGCACTTGATTTACAAGA | 0.99 | -3.48 | 93.80 |
| *CBL10* R | GGAGCATTTGGACTAAAGATGTG |  |  |  |
| *MYB77* F | TCACAGACATGCTGCTTGAT | 0.87 | -3.56 | 90.94 |
| *MYB77* R | GTAGTTGTTGCTCTCTCCATCTC |  |  |  |
| *ADP* F | GCAGCTGAAATCACCGATAAG | 0.97 | -3.22 | 104.43 |
| *ADP* R | CCAGTCAAGTCCCTCATACAA |  |  |  |
| *ACT* F | CCATGAGACCACCTACAACTC | 0.88 | -3.42 | 96.064 |
| *ACT* R | TCAGCAATACCAGGGAACATAG |  |  |  |

**Supplementary Table S3.** Quality metrics of Unigenes**.**

| Table S3: Quality metrics of Unigenes. Sample | Total Number | Total Length | Mean Length | N50 | N70 | N90 | GC(%) |
| --- | --- | --- | --- | --- | --- | --- | --- |
| C1 | 124476 | 80528211 | 646 | 982 | 524 | 268 | 46.97 |
| C2 | 130593 | 85992148 | 658 | 993 | 544 | 273 | 46.84 |
| C3 | 135411 | 90715457 | 669 | 1021 | 558 | 277 | 46.17 |
| T1 | 103504 | 69030681 | 666 | 1062 | 550 | 268 | 49.18 |
| T2 | 74432 | 59750370 | 802 | 1357 | 764 | 309 | 49.6 |
| T3 | 83363 | 57997568 | 695 | 1083 | 621 | 283 | 50.18 |
| All-Unigene | 242833 | 1.8E+08 | 742 | 1223 | 663 | 289 | 47.15 |
